# CRISPR-Cas-Led Revolution in Diagnosis and Management of Emerging Plant Viruses: New Avenues Toward Food and Nutritional Security

**DOI:** 10.3389/fnut.2021.751512

**Published:** 2021-12-16

**Authors:** Susheel Kumar Sharma, Om Prakash Gupta, Neeta Pathaw, Devender Sharma, Albert Maibam, Parul Sharma, Jyotsana Sanasam, Suhas Gorakh Karkute, Sandeep Kumar, Bijoya Bhattacharjee

**Affiliations:** ^1^ICAR Research Complex for NEH Region, Manipur Centre, Imphal, India; ^2^Division of Quality & Basic Science, ICAR-Indian Institute of Wheat and Barley Research, Karnal, India; ^3^Crop Improvement Division, ICAR-Vivekananda Parvatiya Krishi Anusandhan Sansthan, Almora, India; ^4^Department of Plant Breeding and Genetics, Punjab Agricultural University, Ludhiana, India; ^5^Division of Crop Improvement, ICAR-Indian Institute of Vegetable Research, Varanasi, India; ^6^Department of Plant Pathology, Odisha University of Agriculture & Technology, Bhubaneswar, India; ^7^ICAR Research Complex for NEH Region, Umiam, India

**Keywords:** CRISPR-Cas, viral resistance, genome editing, diagnostics, management, CRISPR regulatory framework, food and nutritional security

## Abstract

Plant viruses pose a serious threat to agricultural production systems worldwide. The world's population is expected to reach the 10-billion mark by 2057. Under the scenario of declining cultivable land and challenges posed by rapidly emerging and re-emerging plant pathogens, conventional strategies could not accomplish the target of keeping pace with increasing global food demand. Gene-editing techniques have recently come up as promising options to enable precise changes in genomes with greater efficiency to achieve the target of higher crop productivity. Of genome engineering tools, clustered regularly interspaced short palindromic repeats (CRISPR)/CRISPR-associated (Cas) proteins have gained much popularity, owing to their simplicity, reproducibility, and applicability in a wide range of species. Also, the application of different Cas proteins, such as Cas12a, Cas13a, and Cas9 nucleases, has enabled the development of more robust strategies for the engineering of antiviral mechanisms in many plant species. Recent studies have revealed the use of various CRISPR-Cas systems to either directly target a viral gene or modify a host genome to develop viral resistance in plants. This review provides a comprehensive record of the use of the CRISPR-Cas system in the development of antiviral resistance in plants and discusses its applications in the overall enhancement of productivity and nutritional landscape of cultivated plant species. Furthermore, the utility of this technique for the detection of various plant viruses could enable affordable and precise in-field or on-site detection. The futuristic potential of CRISPR-Cas technologies and possible challenges with their use and application are highlighted. Finally, the future of CRISPR-Cas in sustainable management of viral diseases, and its practical utility and regulatory guidelines in different parts of the globe are discussed systematically.

## Introduction

### Challenges to Global Food and Nutritional Security and Overview of Plant Disease Resistance as a Multicomponent System

It has been projected that the world human population will reach the 10-billion mark by the year 2057. The current average population increase is estimated at 81 million people per year (1). To meet the ever-increasing population's growing food and nutritional requirements, concerted efforts are needed to intensify food production and increase the nutritional value of foods produced (2). Many factors, such as abiotic and biotic stresses and climatic disruptions, pose significant challenges to food production. Up to 40% of crop yields are lost to pests and diseases worldwide (3). The plant diseases caused by fungi, bacteria, viruses, and nematodes largely burden the global agricultural production in general and food and nutritional security in particular. Among these pathogens, viruses and viroids account for losses of up to 100% in case of severe infections depending on the crop species (4). Due to their unique nature and infection cycle, viruses are considered the most challenging to manage among all plant pathogens. With an array of new infections and novel viruses identified using new tools and techniques, the past few decades have witnessed the emergence of viruses as a severe and challenging threat to agriculture worldwide, amounting to losses of several billion dollars annually (5). Recently, owing to erratic climatic disruptions, there have been several reports on the emergence and re-emergence of viruses, the reason for which ranges from modified cropping practices (monocropping and introduction of monoculture of new crops in different geographical areas), free global trade, to the introduction of infected germplasm coupled with the ability of viruses to rapidly evolve and adapt (6). In response to infections, plants, over the years, have developed a complex and intricate defense mechanism, enabling them to avoid, suppress, or defend against a range of pathogens. A plant defense mechanism relies on pathogen recognition followed by the induction of signaling mechanisms leading to resistance or defense. Plant immunity is analogous with the immunity activation component (IAC) associated with the recognition of molecular patterns, i.e., microbe-/pathogen-associated molecular patterns (MAMPs/PAMPs) or damage-associated molecular patterns (DAMPs), effectors, and the immunity modulation component (IMC) that deals with the regulation of immune response.

Plant viruses, in general, have a narrow host range, and the number of non-host plant species far exceeds that of the host ones. In hosts plants, viruses encounter several defense mechanisms; while some act against all viruses, others are specific to a virus and involve R (resistance) genes. In many cases, R genes do not necessarily confer total resistance where virus replication is observed, albeit in a low titer (7) and, thereby, confer only partial immunity. Non-host resistance is a general, non-specific resistance involving two types of mechanisms (8). While type 1 is associated with the activation of the basal defense mechanism, like modulation of the cell wall or activation of secondary metabolite production, type 2 is associated with necrotic lesions and is induced after subduing type 1 infection. Type 2 mechanism is associated with the recognition of molecular patterns like MAMP/PAMPs, and activation of PAMP-triggered immunity (PTI) responses (9). Plant viruses also encounter an antiviral silencing barrier of the host known as RNA interference (RNAi) as a first response to infection, where double-stranded (ds) RNA triggers the silencing of viral genes *via* the action of proteins involved in an RNA-induced silencing complex (RISC). Therefore, dsRNA acts as a MAMP/PAMP for an RNAi acting as a PTI. RNA silencing also includes a regulatory mechanism involving the host-encoded micro-RNAs (miRNAs). While fungal and bacterial diseases are generally managed using antifungal or antibacterial agents, the specific and daunting task of viral disease management relies more on preventing viral infection in plants or developing virus-resistant plants using various strategies developed specifically for a particular target virus. Among these strategies, the use of genomic tools holds utmost importance in terms of robustness and specificity. In addition, accurate diagnosis is the first and most essential tool to identify and characterize a virus infection in plants, thereby paving the way for their downstream management.

### The Emergence of Virus-Led Epiphytotics and Associated Challenges to Global Food Security

Evolution and diversification in plant pathogens lead to the emergence and re-emergence of new diseases in different cropping systems and geographical areas. Viruses have either DNA or RNA as genetic materials and lack proofreading and correction mechanisms during the replication of their genetic material (particularly in the case of RNA viruses). Frequent occurrences of recombination and mutation lead to the emergence of new variants and strains of viruses (10). It is, however, difficult to describe the exact origin of virus variants, as they co-evolve along with their respective hosts coupled with crop domestication, introduction, and diversification (11). Variation occurs continuously in viruses and their vectors in the adaptation time scale against selection pressure posed by host resistance and immunity (11). Continuous evolution in plant viruses, coupled with frequent occurrence of mixed infections of taxonomically different viruses in the same host, leads to the development of new virulent species/strains, thus reducing host resistance durability. With the advent of technologies such as deep sequencing, the detection and characterization of new and emerging virus variants and species have seen major upsurge during the last 2 decades at the global level. Agricultural systems have, thus, witnessed the emergence of new viruses and their variants, and earlier reported viruses of minor economic significance are now becoming causes of major epiphytotics. Some classical examples of virus-led epiphytotics are discussed here.

Infection of maize streak virus (MSV), a mastrevirus complex having 11 strains reported from different parts of the world that could infect more than 80 species in the *Poaceae* family, is a significant constraint to maize production across the globe and can cause losses of up to 100% (12). Complex genotypic structure and rapidly evolving MSV population make it challenging to manage, although host resistance has been well-worked out particularly in Africa (12, 13). Single infection of High Plains wheat mosaic virus (HPWMoV), an emaravirus, and its co-infection with wheat streak mosaic virus (WSMV) in wheat have posed a serious challenge to this staple food (14). In pulses, yellow mosaic disease of mungbean, urdbean, and soybean caused by a begomovirus complex is considered a significant threat (15). The pigeonpea sterility mosaic virus (PPSMV), having five segmented RNA genome has emerged as a major threat to pigeonpea production during the recent two decades (16). Cotton leaf curl disease (CLCuD), caused by genetically distinct virulent strains of begomoviruses, has led to the occurrence of multiple epiphytotics in major cotton growing parts of India, Pakistan, China, and the United States (17). The leaf curl resistance developed by the introgression of two genes in cotton cultivars was broken due to the evolution of recombinant resistance-breaking strain, cotton leaf curl Burewala virus (CLCuBuV) in Pakistan and Indian cotton-growing belts (17–19). Interestingly, resistance-breaking strains quickly replaced the earlier strains and caused havoc to cotton production in this region.

Perennial fruit crop species are infected by several viruses. The citrus tristeza virus (CTV) infection has always remained a major challenge for more than a century due to its complex genetic structure and evolving strains (20). Escape of its detection during initial years in quarantine system, introduction and spread of infected planting materials (rootstocks, grafted trees, and scions), efficient insect vectors, and rapidly evolving CTV genetic variants have made it a virus of global importance. Grapevine being a vegetatively propagated fruit plant has been identified as a sink of the plethora of viruses. Infection of 23 viruses and viroids was identified just from three cultivars (21). Widespread occurrence of grapevine leafroll-associated virus (GLRaV)−3 and −2, grapevine rupestris stem pitting-associated virus (GRSPaV), and hop stunt viroid (HSVd) has emerged in grapevines across the globe (22, 23). In bananas, a group of genetically diverse variants of badnaviruses causing streak disease (BSVs) and banana bunchy top virus (BBTV) are the major threat to its production globally (24–27).

Infection of novel virus species and their variants in apple fruit plants was recorded from different apple-growing parts of the world (28, 29). The genetic variants of apple chlorotic leaf spot virus (ACLSV) and apple stem pitting virus (ASPV) cause the devastating ring-shaped rust and green crinkle disease of fruits, respectively (28). Apple necrotic mosaic virus (ApNMV) and apple hammerhead viroid (AHVd) were recently reported in Indian apple groves (30, 31). Similarly, the infection of multiple viruses in pome and stone fruit crops has emerged in the recent decade. Plum pox virus (PPV), transmitted by various species of aphid vectors and infected propagating materials, has emerged as a significant threat in Europe and Asia (32). A similar threat by viruses of different taxonomic groups was reported in apricot, nectarine, plum, cherry, almond, etc. The symptomless decline is caused by a raspberry bushy dwarf virus (RBDV), and blueberry shock virus (BlShV) infection is horizontally transmitted in berries, thus making it difficult to control and could emerge as a major threat (33, 34). Vegetable and spice crops worldwide are severely affected by the emergence of viral infection. Widespread infection of begomoviruses, particularly tomato yellow leaf curl virus (TYLCV), begomovirus complex in tomato, and begomoviruses, potyviruses in chili has been the ever-emerging constraint in these crops due to the evolution and spread of highly virulent genetic variants (35). They were earlier more prevalent in tropical and subtropical regions but now spreading to other temperate areas. Thrips-transmitted tospoviruses, like tomato spotted wilt virus (TSWV), groundnut bud necrosis virus (GBNV), and capsicum chlorosis virus (CaCV), infecting diverse crops of different plant families, have drawn a concern (36). Cucurbit production is hampered worldwide by several virus diseases caused by potyviruses, cucumoviruses, and other virus groups. Cucumber vein yellowing virus (CVYV) has caused significant havoc in the Middle East and Mediterranean regions (37). The production of cassava, an important staple food for a considerable section of the population in Africa and different parts of other continents, is affected mainly by the evolving begomovirus complex causing cassava mosaic disease (CMD). The spread of CMD-associated begomoviruses (African cassava mosaic virus, Indian cassava mosaic virus, and Sri Lankan cassava mosaic virus) through whiteflies and infected planting materials has caused severe losses in its production (38).

The occurrence, evolution, and emergence of infectious, highly virulent, and pathogenically distinct variants of viruses infecting crops of economic importance have put forth a major burden on global food and nutritional security. The plant host-virus-vector continuum presents a unique combination in the ecosystem, where all three components are continuously evolving under varying natural and posed selection pressure. These reports and experiences of working with viruses showed that dynamically evolving practices of mixed farming, introduction and establishment of new host genotypes/varieties to new geographical pockets, and parallel evolution of vector biotypes put unique synergistic effects on viruses to evolve to more virulent species and strains with higher fitness efficiency in the ecosystem.

An array of genomic tools were invented and utilized to effectively combat the emerging virus and virus-like pathogens associated with epiphytotics. Conventional resistance breeding has seen a paradigm shift to genome-assisted breeding and genetic engineering. The latter involves the wide application of RNAi and virus-derived resistance in the last two decades. These techniques, as a whole, are discussed in brief under the heading “Pre-Genome Editing Era” in this review. In the last decade, virus disease management witnessed a significant shift from the pre-genome editing era to the genome-editing era, where several highly efficient genome editing tools are employed. The subsequent section “Genome Editing Era” of this review discusses the details of genome editing tools and their applications with particular reference to plant virology. We also briefly discuss the parallel applications of genome editing in improving yield and quality that could benefit the global population. The review will provide a holistic view on utilizing and combining the genomic editing technologies to fight the emerging viruses simultaneously while fulfilling the overall goal of food and nutritional security.

## Pre-Genome Editing ERA

### Plant Breeding and Genomic Techniques for Developing Disease-Resistant Plants

**Figure 1:**
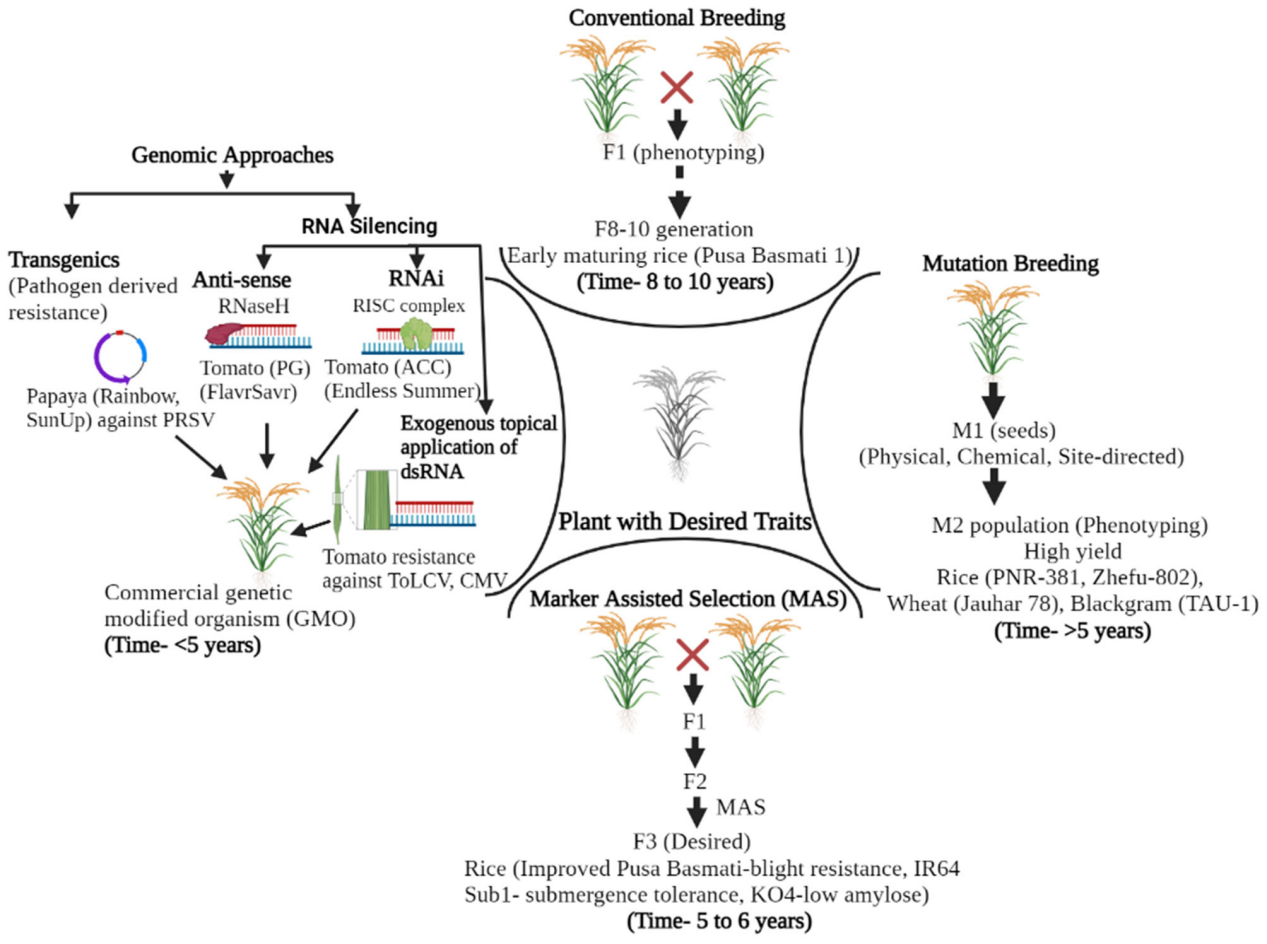
Evolution and application of different tools for crop improvement and disease resistance in the pre-genome editing era. Conventional breeding involves genetic hybridization, rigorous phenotyping, and fixation of segregants and takes 8–10 years to develop a product. These were further revolutionized by marker-assisted breeding wherein foreground and background selection can be performed in early segregating generations (F3), thus reducing the time required for final product development. Mutation breeding was also performed in parallel, although with limited success. Genomic approaches (pathogen-derived resistance, anti-sense technology, and RNAi) are the latest developments in the pre-genome editing era. The exogenous application of virus-derived double-stranded RNA is a novel non-genetically modified (non-GMO) approach in plant virus disease management. The time mentioned for the development of the product through different methods in the Figure is of generalized nature (taken from the reference of annual plant species) and may vary for other plant species and traits concerned.

Several management practices were employed to tackle the losses caused by plant pathogens in different agricultural and horticultural crops; genetic resistance signifies the utmost economical tactic among them. The breakthrough in the field of disease resistance came with the understanding of the gene for gene hypothesis (39, 40). Since then, conventional breeding approaches have comprehensively improved plant cultivars and imparted resistance to plant diseases. The drawbacks of cultural practices and chemical control put the concept of resistant varieties on a better front, and plant varieties with genes resistant to viral infections were utilized as the most sustainable route for their management (41, 42). For now, several dominant and recessive genes involved in viral resistance have been identified and isolated for further deployment in viral resistance programs (7, 43). However, with traditional resistance breeding approaches alone, it is challenging for researchers to keep pace with the reckless evolutionary potential of plant viruses and the increasing demand for the development of disease-resistant varieties. In addition, drawbacks, like non-specificity and being time-consuming and laborious, of these technologies make it challenging to develop economically efficient disease-resistant plants at a pace to tackle the evolving plant viruses (Figure 1). Brief illustrations of different methods in the pre-genome editing era and their attributes are presented in Figure 1, Table 1. Mutation breeding, with limited success, was also employed for imparting desired traits in plant species.

**Table 1:** Different plant breeding techniques used to develop virus-resistant plants and their drawbacks.

**Techniques**	**Examples**	**Drawbacks**	**References**
Gene introgression: Transfer of resistance genes into susceptible host species from wild species	Potato virus X (PVX), potato virus Y (PVY) and potato leaf roll virus (PLRV) in *Solanum tuberosum*	• Incompatibility between species due to ploidy level and endosperm balance number • Pre and post fertilization barriers	(44)
Wide hybridization through bridging species	PLRV, PVY and PVX from the non-tuberous *S. brevidens* into *S. tuberosum*	• Time-consuming • Low efficiency • Resources demanding	(45, 46)
Mutation breeding Involving physical and chemical mutagens	Mungbean yellow mosaic virus (MYMV) in mungbean and soybean	• Less frequency of desirable mutations • Mostly recessive in nature • Pleiotropic effects	(47, 48)
Meristem-Tip Culture *In vitro* culture of shoot tip/apex from mother plant to eradicate viruses associated with phloem	• Sugarcane yellow leaf virus (SCYLV) in sugarcane • Peanut stripe virus (PStV) in patchouli • Piper yellow mottle virus (PYMoV) in black pepper • Sugarcane mosaic virus (SCMV), chlorotic streak disease, white leaf disease	• Costly approach • Problem of acclimatization • Development of variability • Cultural contamination	(49–52)
Somatic hybridization via protoplast fusion	PLRV, PVY, and PVX from *S. brevidens* into *S. tuberosum* hybrids	• Identification problem • Genetic instability	(53–55)
Marker assisted breeding	• Tobacco mosaic virus (TMV) and bamboo mosaic virus (BMV) in tobacco • Rice yellow mottle virus (RYMV) • Barley yellow mosaic virus (BaYMV) in winter barley crops • Soybean mosaic virus (SMV)	• High cost • Low reliability	(56–58)
**Genetic engineering**			
Pathogen derived resistance	• Tobacco mosaic virus (TMV) • Plum pox virus (PPV)	Legislation problems related to biosafety issues	(59, 60)
RNA silencing	• Papaya ringspot virus (PRSV) • Maize streak virus (MSV) • Banana bunchy top virus (BBTV) • Tomato yellow leaf curl virus (TYLCV))	Difficulty in evaluating resistance efficiency	(61–64)
Cross protection	Papaya ringspot virus (PRSV)	Exact molecular mechanism is unclear	(65)

The introduction of molecular markers during the 1980s and 1990s has opened new vistas in crop improvement in general and resistance breeding in particular. The progress in DNA molecular markers and the unceasing advancement of molecular techniques have directed the innovation of marker-assisted breeding (MAB). Furthermore, recombinant DNA technology helped transfer foreign DNA into a host through direct gene transfer (physical, chemical, and electrical methods) and indirect methods, which involves *Agrobacterium* as a biological vector (Figure 1). Transgenic approaches have been used against viral diseases in many crops like tomato, potato, rice, legumes, cucurbits, and others (66) (Table 1).

After that, several viral protein-coding genes, such as replicase (Rep), movement protein (MP), and proteases, were employed for pathogen-derived resistance (PDR) (67–69), but the coat protein (CP) approach is preferred over other techniques due to the durability of protection (70). In 1986, Beachy's lab piloted a revolutionary study on coat-protein-mediated resistance against tobacco mosaic virus (TMV) (59), employing the concept of PDR (71). The transgenic tobacco expressing the coat protein prevented the assembly of TMV (59, 72). The successful attempt on the utilization of CP-mediated resistance was also replicated against potato virus X (PVX), alfalfa mosaic virus (AMV), cucumber mosaic virus (CMV), and tobacco rattle virus (TRV) (72, 73). Similarly, reports on the development of virus-resistant plants using genes like the dominant *SX-5* gene in *Solanum* sp. resulted in tomato spotted wilt virus-resistant plants (74). Cosson et al. (75) stated that proteins encoded by dominant resistance genes like *RTM1* and *RTM2* were involved in suppressing the movement of tobacco etch virus (TEV) in several genotypes of *Arabidopsis*. The recessive genes *rym4/5* in *Hordeum vulgare* were reported to confer resistance to barley yellow mosaic virus (BaYMY) (76). Hart and Griffiths (77) highlighted the association of the *bc-3* gene of *Phaseolus vulgaris* in exhibiting resistance to clover yellow vein virus (CYVV).

Although the transgenic approach has yielded promising results in conferring virus resistance in plants, strict regulatory guidelines for the commercial cultivation and instability of the transgene were the associated limitations (Figure 1). Hence, only a limited area is cultivated under genetically modified (GM) crops (190.4 million hectares) (78). Classical examples of commercial GM crops include the Rainbow and Sun-Up varieties of papaya against papaya ringspot virus (PRSV) and GM squash variety against three different mosaic viruses that were released in the United States (14, 79, 80) (Table 1, Figure 1). The transgenic approach provides durable resistance but is not widely accepted due to apprehensions of adverse effects on untargeted organisms, interference with the function of other essential genes due to insertion of transgenes, being costly, and other regulatory issues. Recently, RNA silencing (RNAi or post-transcriptional gene silencing: PTGS) has evolved as a practical measure against viral diseases. RNA silencing leads to antiviral defense in plants in response to virus infections (81). RNAi is triggered by dsRNA, resulting in high efficiency of gene silencing through specific RNA degradation (82) (Figure 1, Table 1). So far, RNA silencing technology has successfully been applied to target over 60 species of economically important plant viruses. In the bean common mosaic virus (BCMV), two genomic regions (NIb and CP) were targeted, which induced a protection level of about 85–92% in *N. benthamiana* and cowpea through RNAi-inducing dsRNA molecules (83). RNAi-inducing constructs targeting the CP coding region of plum pox virus (PPV) (84), P3 coding region of soybean mosaic virus (SMV) (85), CP coding region of sorghum mosaic virus (SrMV) (86), and proteinase co-factor coding region of cowpea severe mosaic virus (CPSMV) (87) have been used successfully to develop transgene-free virus-resistant plants. MicroRNA (miRNA)-guided silencing was also performed to control virus infection in plants (88). Jiang et al. (89) reported that miRNAs regulated the defense system in *Nicotiana benthamiana* upon co-infection of tobacco curly shoot virus (TbCSV) and its betasatellite (TbCSB). In rice, the expression of miR319 targeting the *TCP21* gene positively acts as plant defense against rice ragged stunt virus (RRSV) infection (90), while in cotton plants, the symptom expression of cotton leafroll dwarf virus (CLRDV) was correlated with the downregulation of specific miRNAs (91). Although these approaches were successful in various plant virus-host combinations, issues of being laborious, time-consuming, expensive, and associated public acceptance hindered their wide popularity and acceptability (Figure 1, Table 1). The exogenous application of naked dsDNA, proven to trigger the RNA silencing pathway against pathogenic viruses, was then attempted (92–94). This dsRNA application approach, although non-transgenic, also has the limitation of having a short virus protection window of only 5 days post application. Most dsRNA-based strategies have been principally limited to either the laboratory stages or restricted field trials (Figure 1). Recently, the topical application of clay-based delivery of pathogen-specific dsRNA has given an affirmative inflection point toward RNAi. The virus-specific dsRNA coated with layered double hydroxide clay nanosheets was successfully employed for virus protection by spraying on the plants and providing prolonged protection. With just a single application of bioclay, the plants were protected from cucumber mosaic virus (CMV) infection in cowpea, and pepper mild mottle virus (PMMoV) infection in tobacco. Recent work in different laboratories to prolong the durability of dsRNA using nano bioclay and their field applications for achieving durable protection against plant viruses has opened new avenues. This approach of translating bioclay-based dsRNAs for viral disease control seems to have a massive potential in the future.

## Genome-Editing ERA

**Figure 2:**
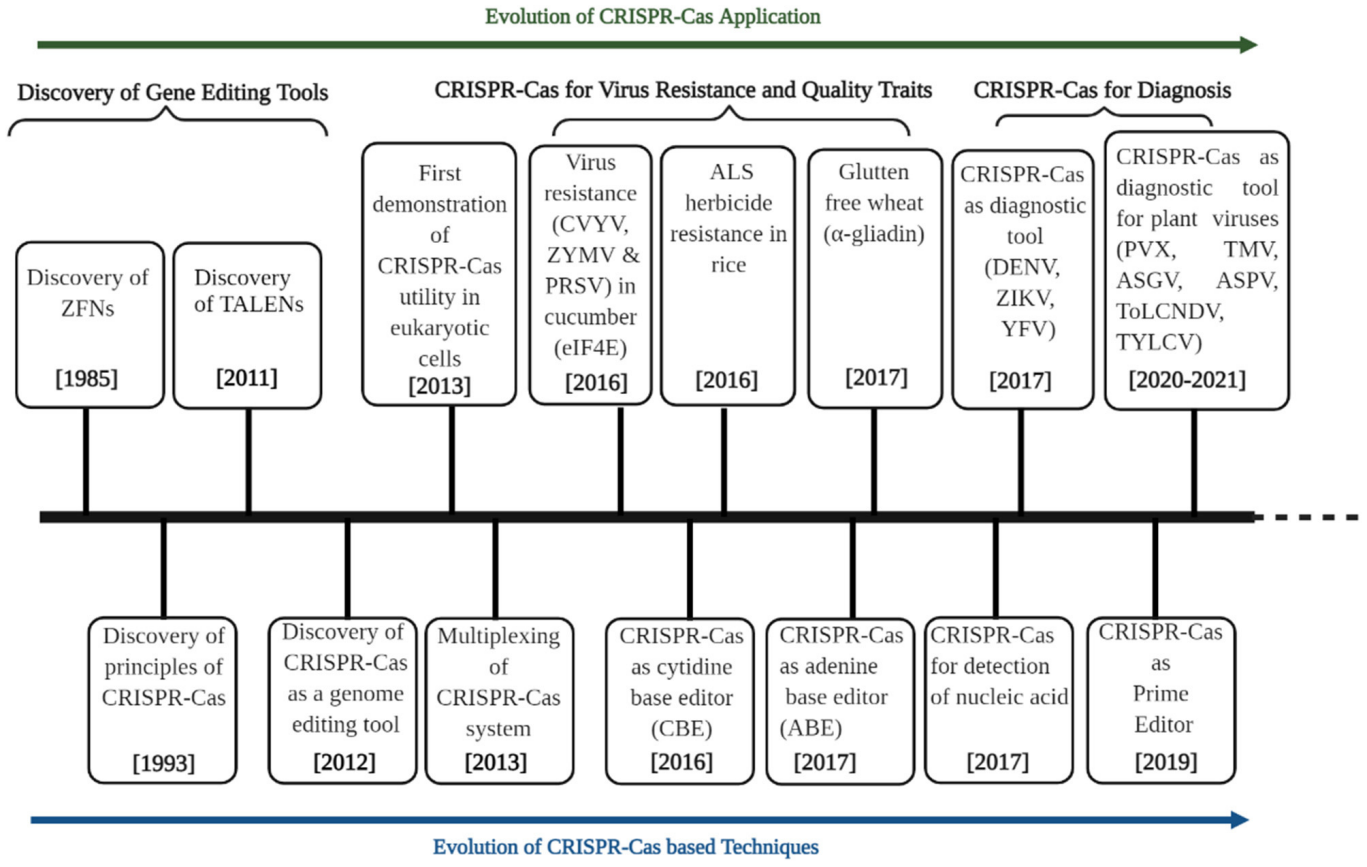
Timeline highlighting the discovery of genome editing technologies (GETs) and their applications in agriculture with particular reference to plant virology and crop improvement. The development of genome editing technologies is divided into three sections: discovery of GETs, applications in virus resistance and quality traits, and diagnosis with a particular focus on using the CRISPR-Cas system. An array of GETs was invented and evolved from 1985 to 2012. The last decade (2013 onward) witnessed a paradigm evolution in CRISPR-Cas techniques (multiplexing, base editors, and prime editors). It sparked their applications in plant virology (virus resistance, diagnostics) and crop improvement for quality. DENV, Dengue virus; ZIKV, Zika virus; YFV, Yellow fever virus; PVX, potato virus X; TMV, tobacco mosaic virus; ASGV, apple stem grooving virus; ASPV, apple stem pitting virus; ToLCNDV, tomato leaf curl New Delhi virus; TYLCV, tomato yellow leaf curl virus.

Genome editing refers to the process of inducing a precise variation in the targeted part of a genome. The tools used for inducing such targeted mutations are known as genome/gene editing techniques (GETs), which offer precise modification in different forms such as insertions and/or deletions (indels) or base substitutions in target sequences (95). Different genome editing tools, such as zinc-finger nucleases (ZFNs), transcription activator-like effector nucleases (TALENs), and clustered regularly interspaced short palindromic repeats/CRISPR-associated protein (CRISPR-Cas) systems were developed (96) (Figure 2). This section of the review focuses on CRISPR-based genome editing and its mechanism and subsequently discusses its applications in detail.

### CRISPR-Cas System: An Efficient Tool for Targeted Gene Editing

The clustered regularly interspaced short palindromic repeats (CRISPR)/CRISPR associated-(Cas) system consists of two parts: CRISPR array and operon of CRISPR-associated (Cas) genes. CRISPR array is a region in the bacterial genome with short and palindromic DNA repeats with spacer DNAs in between. Spacers in the CRISPR array represent the immunological memory of earlier infections (97). Ishino discovered it first in 1987 in *Escherichia coli* as a defense mechanism against viruses, foreign DNA/RNA, and mobile genetic elements (98). However, Mojica took a sincere note of such repeats and named them first as short regularly spaced repeats (SRSRs) and later as CRISPR (99–101). Its function in providing adaptive immunity was also hypothesized by Lander (100) and Mojica et al. (101). Jansen et al. (99) identified Cas genes associated with DNA repeats in prokaryotes. Later discoveries suggested that the proteins expressed by these Cas genes play an important role in tandem with the CRISPR array, in providing immunity (Figure 2). It was found that during the first attack of any foreign viral or plasmid DNA, Cas proteins help in targeting the specific segment of invading DNA to be inserted in the array. A complex of Cas proteins starts surveillance for a specific short 2–4-bp motif in the target DNA molecule, known as a protospacer adjacent motif (PAM). The Cas proteins, when coming across such a distinct motif, introduce a double-strand break (DSB) in the target DNA and release a protospacer segment that gets inserted between the two repeats of the array to become an additional spacer. These DSBs are induced by utilizing site-specific nucleases, and desired modifications get repaired through an error-prone endogenous DSB repair machinery. Among different site-specific nucleases, CRISPR-Cas9 is more appealing, because it can simultaneously modify several plant genes (102–106). There are exceptions where some CRISPR-Cas systems acquire a spacer, i.e., from the RNA transcript of the invading DNA using a reverse transcriptase enzyme encoded in the CRISPR-Cas locus and most often fused to the Cas1 protein. This process of acquiring a specific segment of invading DNA from the CRISPR array is known as adaptation. The Cas1 and Cas2 proteins play a role in the adaptation process. Cas1 protein cleaves the protospacer containing target DNA and CRISPR array, whereas Cas2 provides structural support to the complex. However, in some CRISPR-Cas systems, there is an involvement of additional Cas proteins (107). In the next step, the CRISPR array gets transcribed to a long pre-CRISPR RNA (pre-crRNA), which, after processing, gets converted into smaller mature crRNAs. The crRNAs are then assembled with one or more Cas proteins into CRISPR ribonucleoprotein (crRNP) complexes. The final stage of the CRISPR-Cas-mediated immune response is interference wherein crRNA-directed cleavage of invading viral or plasmid DNAs occurs. In this stage, the crRNA that remains bound to the crRNP acts as the guide to identify the protospacer sequence in the invading viral or plasmid genome. Once the recognition is met, the invading DNA is cleaved and inactivated by a Cas nuclease (107).

Clustered regularly interspaced short palindromic repeats (CRISPR) can be found in both nucleoids and plasmids. A CRISPR locus contains an array of short repeated sequences (21–48 bp) intervened by spacer sequences (26–72 bp) that are often acquired from plasmids and viruses. The natural mechanism of the immune system can be divided into three stages: adaptation, expression, and interference (108). Based on signature protein, there are six types of systems: types I, II, III, IV, V, and VI. Many initial studies were carried out to employ this bacterial immune system in genome editing tools. In the type II system, Cas9 alone can degrade an invading DNA that complements a single guide RNA. The CRISPR-Cas9 type II bacterial immune system came into the limelight in 2005, with the discovery of the extrachromosomal origin of spacer sequence (109). The ability of targeted genome editing of the CRISPR-Cas9 system is due to the structure and conformation of the Cas9 protein. Cas9 is a bilobed protein containing a large recognition lobe (RecA) and a small nuclease lobe (NUC) connected by a helix bridge. The nuclease lobe has two nuclease domains, RuvC and HNH, and a PAM-interacting domain (110). The Cas9-sgRNA complex scans the pairing site between sgRNA and targeted DNA. As it finds the target site, cleavage of RNA-DNA hybrid occurs, HNH is responsible for cleaving the target site, and RuvC cleaves other non-target sites, resulting in double-strand break (DSB). DSB is repaired by a non-homologous end joining (NHEJ) and homology-directed repair (HDR) mechanism, which causes insertion/deletion (INDEL) and frame-shift mutations with just a few base pair (bp) variance, resulting in premature translation termination and loss of function (111, 112). Compared to other genome editing tools like zinc finger nucleases (ZFN) and transcription activator-like effector nucleases (TALENs), CRISPR-Cas9 is easier to multiplex and design the target construct, as it is an RNA-based approach and it does not work in pairs (113) (Figure 2).

Among various genome editing tools, the CRISPR-Cas system is the most popular due to its advantages over other contemporary tools. As much advanced research on the CRISPR-Cas system has been conducted, many modified versions of the CRISPR-Cas system have also come up (Figure 2). Based on the effector nuclease gene's functionality, the CRISPR-Cas system is divided into two classes. Class I includes types I, III, and IV, and Class II includes types II, V, and VI. The main drawback of the CRISPR-Cas system is the off-target issue, which needs to be taken care of *via* off-target detection and high-fidelity editing. Off-target analysis can be performed using *in silico* tools like Cas-OFFinder, Guide-seq, and Digenome-seq (114). To reduce the chances of off-targets in the CRISPR-Cas system, Cas proteins or guides (gRNAs) need to be engineered. In addition to this, improvement of non-specific base editing is also required (e.g., cytosine/adenine base editor) (115). The large size of Cas protein, however, poses limitations in insert gene size for gene delivery system. To address this limitation, lightweight members like Cas14 are considered the best option (116). For efficient delivery, viral vector systems, such as adeno-associated viruses (AAVs), were employed. The range of editable targets can be expanded using PAM variants, as each Cas protein prefers its PAM sequence, e.g., CRISPR-Cas type II recognizes a G-rich sequence, whereas type V recognizes a T-rich sequence, respectively (117). The science of CRISPR-Cas has evolved at a much greater pace. In addition to discovering an array of sequence-specific nucleases, the options of CRISPR-Cas-led multiplex target have sparked its applications in agriculture (113, 118) (Figure 2). The recent discovery of Cas9-based editing tools known as based editors (BEs) and prime editors (PEs) could lead to desired changes in the target genome with DSB and offers a 10–100-fold higher efficiency in obtaining the desired mutations up to single-base-resolution provided for more flexible applications of the CRISPR-Cas system (118, 119) (Figure 2).

To effectively use CRISPR-Cas-based gene-editing tools, information on molecular functions of target genes and genome sequences is a prerequisite. With the revolution of genomics, genomes sequences of many crop species have been deciphered, and genes associated with traits of economic interest have been characterized. Once the function of a gene is identified, it can then be subjected to targeted genome manipulation using the CRISPR-Cas system (120). After modifications are made, it is crucial to identify the edited plants by comparing them to wild types.

### Identification of CRISPR-Cas-Mutated Plants: Techniques and Methodologies

Once a targeted modification is induced in a genetic locus using the CRISPR-Cas system, it is crucial to ascertain the mutants. To quickly detect/identify CRISPR-Cas induced mutations, various molecular methods have been developed, such as enzymatic mismatch cleavage (EMC), high-resolution melting curve analysis (HRMA), modified migration-based heteroduplex mobility assay (HMA), and traditional polymerase chain reaction (PCR) combined with ligation detection reaction (LDR) (121–123). These methods are discussed in the next part of this section.

#### Enzymatic Mismatch Cleavage (EMC)

Enzymatic mismatch cleavage (EMC) is the most widely used technique to confirm site-specific editing in CRISPR-mutated plants. It takes advantage of enzymes that can cleave hetero-duplex DNA at mismatches created by single or multiple nucleotides (122). This method is more suited for larger indels, as its cleavage efficiency is affected by several factors, such as sequence, flanking sequence among two DNA strands, and length of mismatch pairs (122, 124). Furthermore, although it is simple to use, its sensitivity is relatively poor (125), and it cannot discriminate homozygous and heterozygous mutants from wild-type and biallelic mutants, respectively (126). Endonuclease enzymes, T7 endonuclease 1 (T7E1), and surveyor nuclease are mainly utilized in the EMC assay to cleave one or more base pair mismatches in the heteroduplex DNA, and agarose gel electrophoresis may then be used to examine mutations that occur from these minor mismatches (122).

#### High-Resolution Melting Curve Analysis (HRMA)

High-resolution melting curve analysis (HRMA) is a fluorescence-based technique that involves quantitative-PCR (qPCR) amplification of DNA sequences covering around 90–200 bp of the genomic target, fluorescent dye incorporation, and amplicon melt curve study. HRMA analyzes the melting activity of hetero-duplex and homoduplex DNA fragments to determine the melting temperature (Tm) of a specific PCR component and identify the mutant (121). Since the process is non-destructive, the whole procedure of preparing genomic DNA and detecting mutations takes <2 h. HRMA is a simple method and compatible with the high-throughput screening format (96-well-microliter plates). It is fast, unrestrictive, and suitable for detecting low-level chimeric mutants and single nucleotide polymorphisms (SNPs), but it requires special software and is not ideal for broad indel (> 100 bp) detection (121, 123, 127).

#### Multiplex Ligation-Dependent Probe Amplification (MLPA)

In conventional PCR, in conjunction with ligation detection reaction (LDR) assay, two pairs of primers are generally used for each target amplification and visualized by gel electrophoresis. However, since this method relies on indel detection by agarose gel electrophoresis, its sensitivity to detect mutants with just a few base pair genetic variations is limited, while in the amplicon labeling-based method, i.e., multiplex ligation-dependent probe amplification (MLPA), tri-primers (additional universal 6-FAM 5‘-labeled) were utilized for the target amplification and detected by DNA capillary electrophoresis. The MLPA-based method allows for the detection of CRISPR-Cas9-induced on- and off-target mutations (Indel) and naturally occurring mutations. Additionally, an MLPA-based assay can accurately define indel sizes down to 1 bp and handle high throughput analysis (128).

#### Quantitative PCR (qPCR)

Quantitative polymerase chain reaction (qPCR) is a fast, simple, and effective way to detect CRISPR-Cas-induced mutations by amplifying a target locus and sequencing amplified products. The method of using qPCR to differentiate homozygous and heterozygous mutations has been validated in several plant species (129).

#### Whole Genome Sequencing (WGS)

Whole genome sequencing (WGS) is a highly efficient method for detecting many mutations, such as structural variations, large deletions, insertions, duplications, rearrangements, small indels, SNPs, and on- and off-target mutations induced by CRISPR-Cas in various crops (130). In addition, WGS is effective in detecting low-frequency mutations by utilizing high sequencing depth (123).

Other reported methods include restriction fragment length polymorphism (RFLP) (131), PCR based on two primer pairs (132), tracking of indels by decomposition (TIDE) (133), and CRISPR genome analyzer (CRISPR-GA) (134). Some recently developed methods include PCR coupled with ligation detection reaction (PCR-LDR), annealing at critical temperature PCR (ACT-PCR) (135), indel detection by amplicon analysis (IDAA) (136), cleaved amplified polymorphic sequence (CAPS) (137), and mutation site-based specific primer PCR (MSBSP-PCR) (138). However, most of the developed methods are expensive (PCR and qPCR), sensitive, time-consuming (Sanger sequencing, ACT-PCR, and MSBSP-PCR), less accurate (low detection specificity in CAPS), and unable to detect more significant indel mutations (131, 133). Recently developed methods (WGS and MLPA) can detect natural mutations in addition to -on and off-target mutations (139).

## CRISPR-Cas: Its Application in Plant Virus Resistance And Food Security

### CRISPR-Cas for the Development of Virus-Resistant Plants

**Figure 3:**
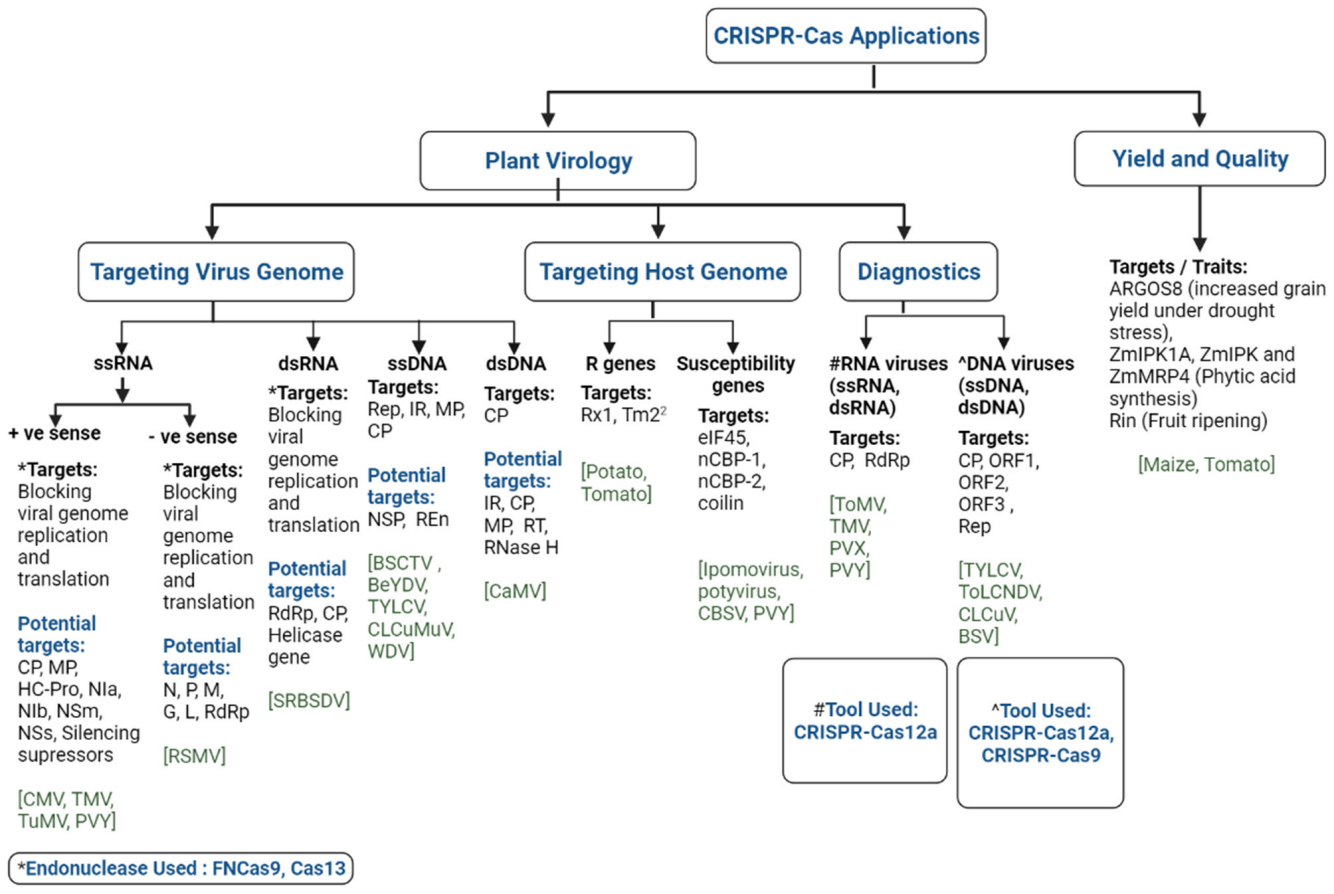
Illustration of the CRISPR-Cas technology and its potential applications in plant virology and food security. Different virus genomes (single stranded-RNA: +ssRNA, -ssRNA; double stranded-RNA: dsRNA; single stranded-DNA: ssDNA; double stranded-DNA: dsDNA) and genomic targets (as evident from a published study) and potential targets are highlighted. The potential targets of (i) +ssRNA viruses [virus families: *Alphaflexiviridae, Bromoviridae, Closteroviridae, Potyviridae, Secoviridae, Solemoviridae*, and *Virgaviridae*]: coat protein (CP), movement protein (MP), helper component-protease (HC-Pro), nuclear inclusion A (NIa), nuclear inclusion B (NIb), cylindrical inclusion (CI), and silencing suppressors; (ii) -ssRNA viruses [virus family: *Rhabdoviridae* (genera: *Alphanucleorhabdovirus, Betanucleorhabdovirus, Cytorhabdovirus, Dichorhavirus*, and *Gammanucleorhabdovirus*)]: nucleoprotein (N), polymerase-associated phosphoprotein (P), putative movement protein (M), viral envelope glycoprotein (G), and RNA-directed RNA polymerase (L) genes; (iii) ssDNA viruses [virus families: *Geminiviridae* and *Nanoviridae*]: replication-associated protein (Rep), intergenic region (IR), MP, CP, nuclear shuttle protein (NSP), replication enhancer protein (REn); (iv) dsDNA viruses [virus family: *Caulimoviridae*]: IR, CP, MP, reverse transcriptase, RNase H. Plant viruses of the *Tospoviridae* family contain three RNA segments [L: -ssRNA, M, and S: ambisense RNA], hence its potential targets [RNA-dependent RNA polymerase: RdRp (complementary sense of L-RNA); Non-structural protein: NSm (genome sense of M-RNA); nucleocapsid: N (complementary sense of S-RNA), non-structural protein: NSs (genome sense of S-RNA)] are presented under the respective +ssRNA and -ssRNA headings in the Figure. The application of endonucleases, FNCas9 and Cas13, was demonstrated for targeting virus genomes. CRISPR-Cas12a and CRISPR-Cas12a, -Cas9 were employed for the specific detection of RNA and DNA viruses, respectively. CMV, cucumber mosaic virus; TMV, tobacco mosaic virus; TuMV, turnip mosaic virus; PVY, potato virus Y; RSMV, rice stripe mosaic virus; SRBSDV, Southern rice black-streaked dwarf virus; BSCTV, beet severe curly top virus; TYLCV, tomato yellow leaf curl virus; CLCuMuV, cotton leaf curl Multan virus; WDV, wheat dwarf virus; CaMV, cauliflower mosaic virus; CBSV, cassava brown streak virus; ToMV, tomato mosaic virus; ToLCNDV, tomato leaf curl New Delhi virus; CLCuV, cotton leaf curl virus; BSV, banana streak virus.

Research efforts on imparting durable resistance to viral infection have recently been reoriented toward genome editing technologies due to their efficiency in creating precise and desired variations in selected loci of plants or viral genomes (118, 120). Among the arrays of genome editing tools, CRISPR-Cas has become more trendy for the development of virus resistance in crops than other tools due to its advantages in terms of targeted genome manipulation and designing (120). The CRISPR-Cas technology has recently been successfully used in many crop species for the engineering of virus-free plants. It is easy to use, has a particular target site of about 20–23 bp, and is easier to predict off-target mutagenesis than RNA-DNA interaction (120, 140). The CRISPR-Cas technique was employed mainly in two ways to develop viral resistance in plants, either by introducing targeted mutations into specific host plants suppressing its susceptibility genes (pro-viral factors) or directly targeting viral genomes (141) (Figure 3). Viruses have either DNA or RNA as genetic material. Around 75% of plant viruses comprise single-stranded RNA (ssRNA) genomes followed by ssDNA and a few double-stranded DNA or RNA viruses (Figure 3). A classical study was conducted to engineer the resistance against DNA viruses, beet severe curly top virus (BSCTV), and bean yellow dwarf virus (BeYDV), in *Nicotiana benthamiana* and *Arabidopsis thaliana* through CRISPR-Cas approach (Table 2) (146, 147). BSCTV- and BeYDV-resistant plants were developed by the sgRNA and Cas9 constructs targeting the coding replication-associated protein (Rep protein) gene and the non-coding intergenic region (IR). Simultaneously, Ali et al. (145) reported on the engineering of sgRNA molecules targeting the intergenic region (IR), coat protein (CP), and metal-binding site involved in protein conformation and DNA cleavage (RCRII) of the bipartite begomovirus, tomato yellow leaf curl virus (TYLCV), in the model plant *N. benthamiana*. The mutant plants showed a reduction in the viral load along with enhanced viral resistance. The generated TYLCV-resistant plants were also reportedly resistant against a mixed infection of monopartite beet curly top virus (BCTV) and bipartite merremia mosaic virus (MeMV) (145). A tobacco plant expressing Cas9 and dual sgRNA targeting two important crucial regions of monopartite cotton leaf curl Multan virus (CLCuMuV) genome (Rep and IR) resulted in complete resistance to the virus infection. Although most studies on the development of resistant plants against geminivirus were reported in model plants, Kis et al. (144) reported the production of wheat dwarf virus (WDV)-resistant barley plants targeting the coding (MP, CP, and Rep) and a non-coding region (IR). In conformity, Tashkandi et al. (149) also reported the development of TYLCV-resistant tomato plants targeting the Rep and CP regions of the viral genome. Although the targeted coding regions in the reported studies yielded promising results, Mehta et al. (161) reported unsuccessful attempts on gaining resistance against African cassava mosaic virus (ACMV) in cassava where conserved single nucleotide mutation led to CRISPR resistance in the edited plants. Such mutations are potentially harmful, as they can lead to the development of more potent and virulent viruses.

**Table 2:** List of virus-resistant plants generated using CRISPR-Cas system.

**Virus genome**	**Plant**	**Target sequences**	**System**	**Mutation**	**Target pathogen**	**Result**	**References**
DNA	*N. benthamiana*	IR and C1(Rep) genes	SpCas9	In-dels	CLCuMuV	Complete resistance to CLCuMuV	(142)
DNA	*Musa* spp.	eBSV Sequence	SpCas9	In-dels	BSV	Inactivation of eBSV gave asymptomatic plants	(143)
DNA	*Hordeum vulgare*	MP, CP, Rep/Rep, IR	SpCas9	Insertion	WDV	No disease symptoms and virus presence	(144)
DNA	*N. benthamiana*	IR, CP, RCRII	SpCas9	In-dels accumulation	TYLCV	No disease symptoms and delayed or reduced virus	(145)
DNA	*N. benthamiana*	Rep/RepA genes, LIR	SpCas9	In-dels	BeYDV	Reduced virus load and symptoms	(146)
DNA	*N. benthamiana* and *Arabidopsis thaliana*	CP, Rep genes, IR	SpCas9	In-dels	BSCTV	Geminivirus-resistant plants of both tobacco and *Arabidopsis*	(147)
DNA	*N. benthamiana*	IR, CP, Rep	SpCas9	In-dels	CLCuKoV, TYLCV, MeMV	Durable resistance to virus infection	(148)
DNA	*N. benthamiana* and *Solanum lycopersicum*	CP, Rep	SpCas9	In-dels	TYLCV	Disease resistance developed	(149)
RNA	*N. benthamiana* and *Arabidopsis thaliana*	ORF1a, ORF2, ORF3, CP and 3'-UTR	FnCas9	No cleavage	CMV	Reduced virus load and symptoms	(150)
RNA	*N. benthamiana*	GFP, HC-Pro and CP	LshCas13a	N.D.	TuMV	Reduced virus load and appearance	(151)
RNA	*Oryza sativa*	Various sequences in SRBSDV and RSMV genomes	LshCas13a	Cleavage	SRBSDV, RSMV	Mild symptoms with reduced viral load	(152)
RNA	*N. benthamiana*	Various sequences in TMV genome	LshCas13a	Cleavage	TMV	Reduced viral load	(152)
RNA	*Solanum tuberosum*	P3,CI, Nib, CP genes	LshCas13a	N.D.	PVY	Resistance to PVY	(153)
RNA	*Solanum lycopersicum*	slDCL2	CRISPR-Cas9	In-dels	ToMV, PVX, TMV	Mutants showed enhanced resistance to virus infection	(154, 155)
RNA	*Cucumis sativus*	*elf4E*/cds region	SpCas9	Deletions	CVYV, ZYMV, PRSV-W	Resistance to CVYV, ZYMV and PRSV-W	(156)
RNA	*Arabidopsis thaliana*	*elf(iso)4E*/cds region	SpCas9	In-dels	TuMV	Potyvirus resistant plants	(157)
RNA	*Manihot esculenta*	*nCBP-1* and *nCBP-2*/cds region	SpCas9	In-dels	CBSV	Reduced virus load and symptoms	(158)
RNA	*Arabidopsis thaliana*	*elf4E*/cds region	SpCas9-cytidine deaminase	Point mutation	CIYVV	Prevent virus accumulation	(159)
RNA	*Solanum tuberosum*	*Coilin gene*	SpCas9	N.D.	PVY	Resistance to virus infection; tolerance to salt and osmotic stress	(160)

*IR, intergenic region; Rep, replicase; LIR, long intergenic region; In-del, insertion and deletion; N.D., not defined; CLCuMuV, cotton leaf curl Multan virus; BSV, banana streak virus; WDV, wheat dwarf virus; TYLCV, tomato yellow leaf curl virus; BeYDV, bean yellow dwarf virus; BSCTV, beet severe curly top virus; CLCuKoV, cotton leaf curl Kokhran virus; CMV, cucumber mosaic virus; TuMV, turnip mosaic virus; SRBSDV, southern rice black-streaked dwarf virus; RSMV, rice stripe mosaic virus; TMV, tobacco mosaic virus; PVY, potato virus Y; ToMV, tomato mosaic virus; PVX, potato virus X; CVYV, cucumber vein yellowing virus; ZYMV, zucchini yellow mosaic virus; PRSV-W, papaya ringspot virus-W; CBSV, cassava brown streak virus; ClYVV, clover yellow vein virus*.

The CRISPR-Cas system has also been effectively employed against several RNA viruses using Cas endonucleases (FNCas9 and Cas13) (Figure 3). Zhang et al. (150) reported the first RNA virus-resistant plants of tobacco and Arabidopsis targeting various regions of cucumber mosaic virus (CMV) and tobacco mosaic virus (TMV) genomes where the mutant plants showed lesser accumulation of the viruses along with reduced symptom expression (Table 2). Concurrently, turnip mosaic virus (TuMV)-resistant tobacco plants edited using the Cas13 system targeting various coding regions (HC-Pro and CP) of the viral genome resulted in reduced viral load and symptom expression (151). Similar attempts to develop resistance to potato virus Y (PVY), rice stripe mosaic virus (RSMV), and Southern rice black-streaked dwarf virus (SRBSDV) have been reported on potato and rice. Therefore, Cas endonucleases (FNCas9 and Cas13) have been proven a powerful tool directly targeting viral RNA in engineered virus-resistant plants but following a transgenic-based approach (Figure 3). However, studies targeting the *eIF4E* gene, also known as the cap binding protein using the Cas9 system, reported transgene-free resistance against RNA-based ipomovirus and potyvirus in cucumber and Arabidopsis, respectively (156, 157). Likewise, Gomez et al. (158) reported resistance against cassava brown streak virus (CBSV) attained by targeting cap-binding protein-1 (nCBP-1) and nCBP-2 through CRISPR-Cas9 in cassava. Similarly, a PVY-resistant potato variety was developed targeting the *Coilin* gene using the CRISPR-Cas9 system, and led to virus resistance and enhanced tolerance to salt and osmotic stress (160). Studies have also reported the utilization of the *Dicer-like2* gene to understand its role in plant defense mechanisms. These studies demonstrated the exciting potential of the CRISPR-Cas system as a powerful tool in developing resistance against plant viruses. The different genomic regions of RNA and DNA viruses that have a role in the infection cycle could be the potential target for CRISPR-Cas-based modification to confer resistance to infection (Figure 3).

### CRISPR-Cas for Plant Virus Disease Diagnosis

Timely, accurate, and sensitive detection of viruses causing diseases in plants is key in their mitigation and management. Lately, there has been tremendous growth in viral disease diagnosis by detecting targeting nucleic acids using CRISPR-Cas based platforms, which are a robust tool compared to other known common diagnostic platforms. The first CRISPR-Cas-based diagnosis was performed using CRISPR-Cas9 endoribonucleases recognizing the double-stranded DNA (dsDNA) (162) (Figure 3). Recent studies reported the use of CRISPR-associated Cas systems viz., Cas12a, Cas13a, and Cas14, for nucleic acid detection (163–165). Gootenberg et al. (164) developed a rapid and sensitive nucleic acid detection method using the CRISPR effector Cas13a combined with the isothermal amplification method named Specific High-Sensitivity Enzymatic Reporter UnLOCKing (SHERLOCK). The developed method could detect DNA or RNA at attomolar concentrations and even with single-base mismatch specificity. The first Cas12a endoribonuclease-based detection method, referred to as DNA endonuclease-targeted CRISPR trans reporter (DETECTR), was used to guide dsDNA targets by crRNA triggering collateral cleavage of short ssDNA carrying a quencher and a fluorophore leading to target recognition *via* generation of fluorescent signal upon target recognition and subsequent reporter cleavage (163). One-HOur Low-cost Multipurpose highly Efficient System (HOLMES) utilizes the Cas-12a effector system combined with loop-mediated isothermal amplification (LAMP) capable of fast and highly sensitive detection of target DNA and RNA (166). In other approaches, a sample is amplified to enrich the target DNA using recombinase polymerase amplification (RPA) reactions or reverse-transcription-recombinase polymerase amplification (RT-RPA) reactions when the target is RNA. The RPA product is then transcribed into RNA using a T7 RNA polymerase. The transcripts obtained are subjected to collateral cleavage with Cas12/13 in the presence of a quenchable reporter ssRNA, and fluorescence is quantified. SHERLOCK, DETECTR, and HOLMES are highly specific and provide attomolar sensitivity in detecting viruses, microorganisms, and transgenes (167–170). The CRISPR-based virus detection shows a vast prospective, but its potential is still not fully utilized. Recent years have witnessed several reports on the development and application of CRISPR-based diagnostics for the robust detection of plant viruses (Table 3, Figure 3). Gootenberg et al. (179) developed a rapid, inexpensive, and sensitive lateral flow “paper strip” test method for application in reliable on-site detection of plant viruses. A one-step-RT-RPA-Cas12a assay for the detection of plant viruses was used (175). The study reports on the development of a one-step *in vitro* Specific CRISPR-based Assay for Nucleic acid detection-one pot (iSCAN-OP) for the diagnosis of potato virus X (PVX) and tobacco mosaic virus (TMV). It includes RT-RPA pre-amplification followed by collateral activity using Cas12a endoribonucleases, subsequent cleavage of the ssDNA reporter molecule, and release of fluorescent signals for quantification. The iSCAN-OP detection assay was combined with a commercially available fluorescence viewer device that enabled a fast and affordable in-field diagnostic platform to detect plant RNA viruses.

**Table 3:** Application of CRISPR-Cas based diagnostics in detection of plant viruses.

**Plant**	**Plant virus/Pathogen**	**CRISPR based diagnostics**	**Targeted genomic region**	**References**
Tobacco	Tomato yellow leaf curl virus (TYLCV), tomato leaf curl New Delhi virus (ToLCNDV)	CRISPR-Cas12a	Coat protein (CP)	(171)
Sugar beet	Beet necrotic yellow vein virus (BNYVV)	CRISPR-Cas12a	RNA-1	(172)
Tomato	Tomato mosaic virus (ToMV)	CRISPR-Cas12a	ORF1	(173)
Tobacco	Cotton leaf curl virus (CLCuV)	CRISPR-Cas9	Rep, βC1	(174)
Tobacco	Tobacco mosaic virus (TMV), potato virus X (PVX), potato virus Y (PVY)	CRISPR-Cas12a	Coat protein (CP)	(175)
Banana	Banana streak virus (BSV)	CRISPR-Cas9	ORF1, ORF2, and ORF3 of BSV	(176)
Apple	Apple necrotic mosaic virus (ApNMV), apple stem pitting virus (ASPV), apple stem grooving virus (ASGV), apple chlorotic leaf spot virus (ACLSV), and apple scar skin viroid (ASSVd)	CRISPR-Cas12a	Coat protein (CP)	(177)
Apple, Pear	Fire Blight (*Erwinia amylovora*)	CRISPR (CR1-CR2-CR3)	T3SS, T3E	(178)
Rice	Rice blast disease (*Magnaporthe oryzae*)	CRISPR-Cas12a	Cry1C	(170)

Similarly, a CRISPR-Cas12a-based visual assay was reported for the field detection of multiple RNA viruses and viroids in apple, i.e., apple stem grooving virus (ASGV), apple necrotic mosaic virus (ApNMV), apple stem pitting virus (ASPV), apple scar skin viroid (ASSVd), and apple chlorotic leaf spot virus (ACLSV) (177). Compared to other detection techniques like RT-qPCR, the CRISPR-Cas12a-RT-RPA platform exhibited higher sensitivity in ASPV and ASGV, detecting 250 copies per reaction to 2,500 copies for ApNMV, ASSVd, and ACLSV, respectively. The CRISPR-Cas12a system was also used for the detection of two begomoviruses, tomato yellow leaf curl virus (TYLCV) and tomato leaf curl New Delhi virus (ToLCNDV) (171). The assay combined LAMP and the CRISPR-Cas12a system to develop a quick and low-cost on-site assay for the diagnosis of TYLCV and ToLCNDV in ~1 h.

Clustered regularly interspaced short palindromic repeat (CRISPR)-based diagnostics within a brief period has evolved from laboratory-based nucleic acid detection tools to the point-of-care or on-site diagnostic tool due to its reliability, cost-effectiveness, and high sensitivity. However, there are still some limitations; one of the major drawbacks is dependence on the pre-amplification step for targets of below femtomolar concentrations. Also, the primers and crRNA designed for the detection assay must be lab tested and standardized (180). Lately, the use of one-step assay combined with easy detection of test subjects has considerably eased the use of CRISPR-based diagnostics. However, using heating devices for higher temperatures and separate steps for sample preparation remains a bottleneck and limits its application for in-field detection of plant viruses. Therefore, overcoming these limitations and further advancement in on-site diagnostic devices linked to the technique will enable additional acceptability of this platform for broader applicability.

### CRISPR-Cas for Enhancing Yield and Quality of Crops

In addition to the field of virus diseases, genome editing techniques, specifically CRISPR-Cas, have brought a parallel revolution in crop improvement programs. The ultimate goal of crop improvement programs is to enhance crop yield and nutritional quality while making them resistant to diseases in order to ensure food and nutritional security and ultimately achieve the sustainable development goal of zero hunger (120, 181–184). The otherwise commonly employed molecular breeding and biotechnological approaches are not efficient in case of challenging to improve traits that demand advanced techniques like targeted genome editing (185, 186). Compared to other breeding practices that usually require 8–10 years, genome editing requires less duration (4–6 years) for product development (187) (Figure 1). Over the past few years, the CRISPR-Cas system has been proven as a game-changing technology in crop improvement programs in many ways, such as mutations in the coding region, promoter editing, gene insertion, prime editing, programmed single base editing, and cell type-specific and conditional mutations (188).

Detrimental environmental factors and climate disruptions primarily affect the yield of crops, and, thus, pose a significant threat to food security. Therefore, it is necessary to develop crops that tolerate environmental stresses without any yield penalty. Crop yield is a complex polygenic trait; thus, it is difficult to improve it by targeting a single gene. Alternatively, targeting regulators of yield-related attributes, such as grain size, grain number, and grain weight, could be a practical approach for a targeted increase in yield (187). Crop yield can also be substantially increased by reducing yield losses caused by environmental stresses. Therefore, genome editing to develop resistance/tolerance to various abiotic stresses will ultimately increase the marketable yield of crops. In the recent past, the CRISPR-Cas system has been successfully implemented in several crops for enhancing yields.

One of the exciting studies involving CRISPR-Cas9-mediated genome editing of multiple genes governing rice yield-related traits resulted in a significant increase in grain yield (189). Four different genes, *Gn1a, DEP1, GS3*, and *IPA1*, which regulate grain number, panicle architecture, grain size, and plant architecture, respectively, were mutated in this study. *Gn1a* mutant plants showed increased plant height, panicle size, and the number of flowers per panicle. In contrast, *DEP1* gene mutant plants exhibited reduced height and short panicles but increased the number of flowers per panicle. The best results were obtained with *GS3* mutants, with a significant increase in grain weight, grain size, and grain length. Mutations in the *IPA1* gene, which defines plant architecture, could result in enhanced plant height and number of flowers per panicle, and reduced tillers as expected, ultimately resulting in increased grain yield. Several other studies have been conducted by targeting yield-related genes in various crops, such as *OsGRF4* for increased grain size and yield in rice (190), *GW5* (191), *OsAAP3* for increased tiller number in rice (192), *TaGASR7* for high grain weight in wheat (193), *ARGOS8* in maize for enhanced grain yield (194), and several other horticultural crops. Tomato has been intensively subjected to genome editing to improve various traits, such as yield, as a model fruit crop. Rodríguez-Leal et al. (195) engineered a tomato fruit crop for quantitative traits related to fruit size, inflorescence branching, and plant architecture, resulting in increased yield. They targeted the genes involved in the classical CLAVATA-WUSCHEL (CLV-WUS) stem cell circuit. In tomatoes, floral organ number and fruit size are inversely proportional to the expression of the *SlCLV3* gene. CRISPR-Cas9-mediated mutations at eight loci in the promoter region of the gene resulted in mutants with increased floral organs and fruit size. Targeting the *COMPOUND INFLORESCENCE (S)* and *SELF PRUNING* (*SP)* genes that govern inflorescence development and indeterminate growth produced bushy determinate plants with excessively branched inflorescences with hundreds of flowers. These classical studies on the use of the CRISPR-Cas9-mediated genome editing technique in improving yield and yield-related traits controlled by multiple genetic loci opened new avenues in crop improvement and can be replicated in other crop plants.

In order to fight the issues of hidden huger on a globe-wide scale, efforts have recently been shifted to focus on enhancing the quality and nutritional content of food grains, vegetables, and fruits, and genome editing technology has served the purpose very effectively (196–198). Among genome-edited foods, the mushroom was the first one to reach the market. The polyphenol oxidase gene in mushrooms was mutated by CRISPR-Cas9 to produce strains with reduced browning traits (199). In rice, starch quality is one of the basic essential quality parameters; thus, CRISPR-Cas9 mediated genome-edited rice with high amylase and low viscosity was developed by knocking out starch-branching enzyme gene *SBEIIb* (200). Besides starch quality, the aroma is a very precious trait determining rice quality due to the presence of 2-acetyl-1-pyrroline in rice grains. Mutations in the *BADH2* gene responsible for the production of g-aminobutyric acid (GABA) result in more production of 2-acetyl-1-pyrroline (201). Similar results were obtained by knocking out the *BADH2* gene in a Zhonghua 11 rice cultivar using CRISPR-Cas9. Thus, elite and high-yielding rice varieties can be modified for increased content of 2-acetyl-1-pyrroline by knocking out the *BADH2* gene using CRISPR-Cas9. Recently, many other traits, such as low cadmium content (202), high oleic content (203), increased β-carotene (204), and red rice (205), have been targeted by CRISPR-Cas9 editing in rice. Recently, using CRISPR-Cas9, a new allele *BADH2* was created in the non-fragment japonica and indica rice varieties NJ1 and HHZ. This was further utilized for grain aroma improvement in three-line hybrid rice (198). Similarly, in wheat, CRISPR-Cas9-led knockout of α-gliadin genes resulted in low gluten-content seeds, which showed an 85% reduction in immunoreactivity (206). The protein content of wheat grains has also been increased in *GW2* knock-out plants (127). Maize has also been subjected to CRISPR-Cas9-mediated genome editing to target the *IPKA1, IPK*, and *MRP4* genes involved in phytic acid synthesis to reduce the content of phytic acid, which is an anti-nutritional component (207).

Oil content and fatty acid composition are some of the most important quality parameters in oilseed crops. Increased oleic acid and decreased linoleic acid content, increased C18 unsaturated fatty acids, and reduced polyunsaturated fatty acids (PUFAs) are the most desirable traits in this context. The CRISPR-Cas9 technique has been successfully implemented by targeting various fatty acid synthesis pathway genes in rapeseed (increased 18:1 and reduced 18:2 fatty acids), camelina (reduced PUFAs, increased 18:1, altered amino acid profile), soybean (increased 18:1 and reduced 18:2), peanut, and pennycress for enhancing the quality and quantity of oil (208).

The CRISPR-Cas system was employed to enhance the quality, shelf life, and functional metabolites of fruits and vegetable crops. Using the CRISPR-Cas9 technology, tomato fruits with high lycopene content were developed, leading to their enhanced quality (209). This was achieved by inhibiting the conversion of lycopene to β- and α-carotene by mutating the *SGR1, LCY-E, BLC, LCY-B1*, and *LCY-B2* genes. Besides lycopene, breeders aim to increase the content of γ-aminobutyric acid (GABA), a neuro-suppressant that acts in blood pressure regulation. CRISPR-Cas9-mediated knock out of the *SlGAD2* and *SlGAD3* genes resulted in 7–15 times increased accumulation of GABA (210). To delay the ripening of tomato fruits to ultimately enhance the shelf life, a ripening inhibitor *(RIN)* gene was knocked out in different tomato cultivars (211). Alternatively, the targeted mutation in long non-coding RNA1459 (*lncRNA1459*) through the CRISPR-Cas system resulted in delayed ripening along with reduced ethylene and higher accumulation of lycopene (212). Similarly, starch quality in potatoes was altered to produce tubers with increased amylopectin by editing the granule-bound starch synthase gene (213). CRISPR-Cas9-led knocking out of the polyphenol oxidase gene reduced enzymatic browning in potatoes (214). In potatoes, steroidal alkaloid α-solanine is considered an anti-nutritional compound; therefore, CRISPR-Cas9-edited potato plants free of α-solanine were produced by targeting the *St16DOX* gene (215). In the same way, tartaric acid, an anti-nutritional factor in grapes, was efficiently reduced by targeting the *IdnDH* gene by CRISPR-Cas9 (112). Bioactive compounds or other nutritionally essential compounds in most crops are synthesized by complex pathways involving many genes. Therefore, manipulation of such pathways for the production of desired compounds needs simultaneous alteration of more than one gene. Recent advancements in multiplexing in the CRISPR-Cas system help to target multiple genes for editing at a time by CRISPR-Cas9, which has made it possible to manipulate such metabolic or biosynthetic pathways. These advancements have opened new avenues not only to target and improve yield and quality-related multigenic traits but also simultaneously develop resistance to biotic and abiotic stresses particularly to combat the most difficult viral pathogens. The CRISPR-Cas technology, with a wide range of applications, therefore, has ample potential in paving ways to achieve food and nutritional security for the benefit of the entire globe.

## Discussion

In the era of climate disruptions, decreasing cultivable lands, and burgeoning human population, which is expected to touch 10 billion within the next few decades, food security remains a major challenge at the global level. To meet food and nutritional demands, various programs targeting nutritional enhancement and increase in crop productivity are being undertaken on a war footing. While achieving higher yields per unit of cultivated land and quality standards of agricultural produce have remained generalized targets globally, emerging concerns raised by climatic disruptions and various biotic and abiotic factors pose a serious hurdle in achieving the targeted growth rate in food production. Plant diseases alone cause around 40% of global crop losses, and their emergence and re-emergence have always remained an alarming concern (216). Recently, a series of new emerging viral diseases in crops, along with rapidly emerging or re-emerging viral pathogens, has caused a global concern, the reason for which ranges from dynamic cropping practices, free global trade, to the introduction of infected germplasm coupled with the ability of viruses to evolve and adapt rapidly (6). The American Phytopathological Society (APS) classified these pathogens into four categories: new, emerging, re-emerging, and threatening, respectively. In the past, geminiviruses, potyviruses, and some new groups of viruses were witnessed as major viral pathogens associated with diseases in tomatoes, cotton, melons, lettuce, beans, and other crops. The emergence or re-emergence of pathogens is a concern because these newly evolved pathogens can infect new crops in the vicinity and cause epiphytotic. Sharma et al. (217) reported the infection of a new virus (Large cardamom chirke virus: macluravirus) in chili, which is the result of its natural host shift from large cardamom plantations (218). Such events of host shift cause major impediments to crop productivity and affect food security. Therefore, to tackle these, newer and more efficient techniques are utilized across the world. However, conventional breeding along with molecular techniques have been used for increasing crop production in the past. Recently, the advent of gene editing techniques has opened new avenues for a better outcome. The CRISPR-Cas system, among gene-editing techniques, is the most powerful tool to achieve the targeted editing in genomes through faster and precise means.

The CRISPR-Cas system was first used to detect the Zika virus in humans (164). This tool has evolved over the past years for the precise and cost-effective detection of various plant viruses. The technique has been used to detect a single pathogen and multiplexed to diagnose multiple viruses at a time (179). The recent development of tools like SHERLOCK, DETECTR, and HOLMES, which are highly specific and sensitive in detecting viruses, has opened new horizons in virus diagnostics (167–170) (Figure 3). The CRISPR-Cas system has also been used for in-field and on-site detection of plant viruses (179) along with the development of a one-step assay (iSCAN-OP) (175). A wide range of wheat, tomato, cassava, potato, rice, and cucumber plants was developed through CRISPR-Cas engineering, with the target of imparting disease resistance mainly to plant viruses (144, 149, 156–158).

Due to its simplicity and applicability, CRISPR-Cas tools have been widely used in crop improvement programs *via* mutations in codon region, promoter editing, gene insertion, prime editing, programmed single base editing, and cell type-specific and conditional mutations (188). In order to diversify the nutritional and functional traits of crops controlled by polygenes, genes and their controlled traits associated with the metabolic pathway need to be investigated for successful trait improvement without undesirable pleiotropic defects. For instance, targeting multiple genes governing yield-related traits in rice regulating grain number, panicle architecture, grain size, and plant architecture through the CRISPR-Cas system resulted in a significant increase in grain yield, thereby improving yield and yield-related traits (189). Similar studies on genes related to the yield trait in wheat (219) and maize for enhanced grain yield (194) have also been successfully reported. Concurrently, this technique has also been utilized to enhance the quality of starch in rice along with enhancement of aroma (200, 201), low cadmium content (202), increased β-carotene (204), etc., thereby producing elite and high-yielding rice varieties. Apart from enhancing the nutritional aspect, CRISPR-Cas has also been utilized to reduce the impact of anti-nutritional factors in certain crops like maize and grapes (112, 207). Recent advancements in the development of base editors and primer editors offer more exciting options and robustness in the efficiency of the CRISPR-Cas system (118, 119).

The futuristic and potential use of the CRISPR-Cas technique lies in targeted editing of pro-viral (susceptibility) genes having a positive role in the virus infection cycle. The multiplexing in the CRISPR-Cas system has now enabled to target of multiple genes in a single approach and develop end products encompassing more efficient desirable traits like yield, quality, and resistance to disease. In a recent study, a transient assay was performed by multiplexing of gRNA-Cas9 modules in the model plant *N. benthamiana*, and showed a high degree of resistance to chili leaf curl virus (ChiLCV) and restriction of the generation of escape mutants as compared to single sgRNA (220). A similar study on targeting multiple overlapping viral genes of cotton leaf curl virus (CLCuV) resulted in better interference with virus proliferation (221). A schematic representation of different usage of the CRISPR-Cas tool indicates its versatile applications right from precise genome editing to diagnosis within a short period (Figures 2, 3). However, efficient multiplexing, reduced off-targets, and designing on-site diagnostic devices remain the main bottlenecks that need to be addressed with advancement in the field thereby garnering its broader applicability. The coming years are expected to witness the exciting widespread applications of CRISPR-Cas-based multiplexing and specific targeting of genomes up to a single-base-resolution using base editors and prime editors to fight the menace of viral infections in plants and achieve the ultimate goal of food and nutritional security. The merits of this technique far outweigh the demerits. However, the differential regulatory guidelines and general acceptability of CRISPR-Cas-engineered crops are a hurdle that has to be dealt with comprehensive, collaborative plans between policymakers and researchers for the integration of CRISPR-Cas-based products to achieve global food and nutritional security. These points are discussed in detail in the subsequent section of the review.

### Future Prospective and Challenges for CRISPR-Edited Crops

#### Regulations for CRISPR-Edited Plants: A Global and Indian Scenario

During the CRISPR-based genome editing process, several bases with large segments are removed or interchanged (222). There are three classes of genome editing employing site-directed nuclease SDNs: (i) induction of single point mutations or InDels (SDN-1), (ii) editing of a few base-pairs with an external DNA template sequence (SDN-2), and (iii) insertion of longer strands (SDN-3) of transgenes or cisgenes. The most recent advancement in genome editing is base editors, which require the combination of non-cutting Cas9 with deaminase nucleotide, leading to the point mutation of A/T base pairs into C/G vice-versa without cleaving the genome (166, 219, 223), which comes under the SDN-1 class.

**Figure 4:**
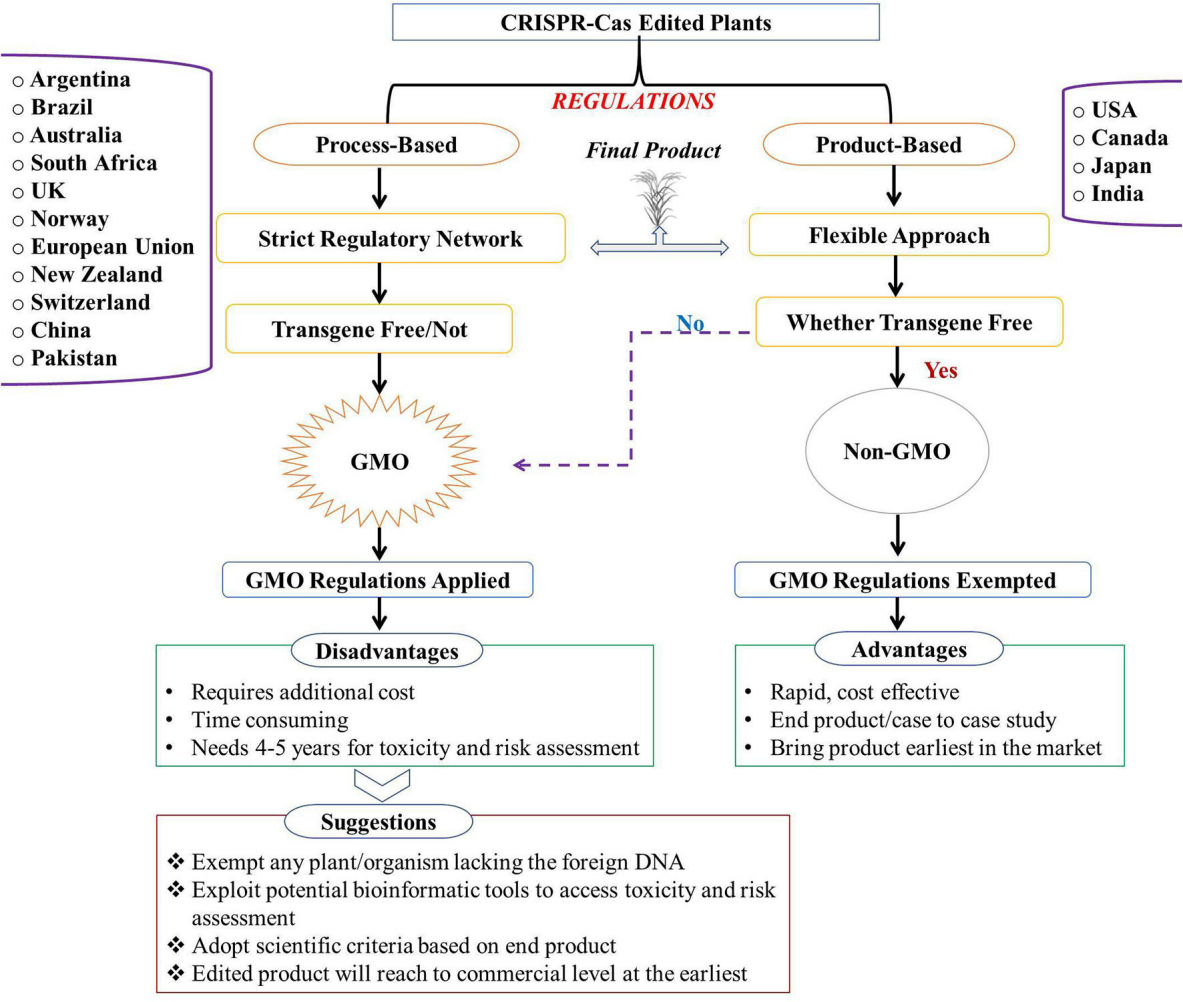
Regulatory aspects of genome-edited (CRISPR-Cas modified) plants at the global level. Different countries are now using one of two regulatory systems. Process-based regulations: countries (Argentina, Brazil, Australia, South Africa, United Kingdom, Norway, European Union, New Zealand, Switzerland, China, and Pakistan) regulate end products based on the process used, which leads to strict GMO regulations. End-product-based regulations: countries (United States, Canada, Japan, and India) regulate the end product based on its attributes. GM, genetically modified; GMO, genetically modified organism [modified from Ahmad et al. (227)].

The Cartagena Protocol on Biosafety created the regulation norms for international trading in living genetically modified organisms (LMOs). However, there are some differing viewpoints on the production, consumption, and regulation of CRISPR-edited plants that have been taken by several countries (224). Essentially, two frameworks are being followed by many nations to regulate CRISPR-edited plants, i.e., (i) regulation of the procedure to generate genome-edited plants and (ii) regulation based on final product attributes (225, 226) (Figure 4). Policies vary among nations, wherein a few countries have exceeded procedures to deal with genome-edited plants or deregulate them, while many have passed the guidelines (228). The United States regulatory policies are mainly based on technical characteristics of modified qualities and their eventual use as an end product (risk-based) (227–229). The Canadian Food Inspection Agency (CFIA) attributed more importance to the product developed rather than the process employed in its development (novelty and risk-based) concerning CRISPR applications (230). Furthermore, Germany and the Netherlands are nearing the completion of rules requiring genome editing crops to be labeled as non-GMO (231). All European Union (EU) member states are working on their national regulatory guidelines, and their regulatory trigger is process-based. Chinese authorities have taken steps to ensure that CRISPR-edited food is managed and risk assessed on a case-by-case basis.

In Australia, the organisms manipulated through SDN-1 are not regulated under GMO regulations, whereas SDN-2 and SDN-3 are regulated under strict GMO regimes (232, 233). In New Zealand, the Hazardous Substances and New Organisms (HSNO) involves a process-based regulatory framework for GMOs (227). As a result, this allows products produced by any genome-editing technology to be regulated within the GMO framework. Additionally, any product developed through classical/induced mutagenesis by chemical/physical mutagen or transgene-free will be evaluated case by case (234). In India, the Department of Biotechnology (DBT) issued draft guidelines for gene editing regulation in 2020, which required additional safety and efficacy testing for genome-edited crops. The guidelines regulate the process used to generate genetically manipulated plants rather than final product attributes. There are no established timelines for the regulatory approval of gene-edited products at present.

From the above discussion, it is clear that regulations governing gene editing are constantly changing worldwide. Therefore, under the Genetic Literacy Project (GLP), an Agriculture Gene Editing Index has been formed to compare the regulatory restrictions from country to country. Ratings on the gene-editing index (Supplementary Table 1) reflect the current state of gene editing regulations and can be accessed from the Global Gene Editing Regulation Tracker [https://crispr-gene-editing-regs-tracker.geneticliteracyproject.org/].

Risk assessment and biosafety issues may be addressed by utilizing the advanced bioinformatics tool to detect the potential off-target effects by comparing the gRNA and dsRNA sequences with the reference genome. The activity of Cas9 in subsequent generations also needs to be addressed (227) (Figure 4). Plasmid-free transformation events are now available to tackle this issue. Virus-based vector systems can overcome these issues but have some limitations of poor editing efficiencies and poor inheritability in the subsequent generations of desired edits. For the future development of Cas9-free plants, these constraints must be addressed. In this case, protoplast-based gRNA and Cas9 complex insertions can be performed. In all crops, efficient chloroplast-based transformation systems are still lacking. These issues will need to be addressed properly if the latest gene-editing tools are to be fully utilized.

### Advantages, Limitations, and Challenges of CRISPR-Based Gene Editing

Clustered regularly interspaced short palindromic repeat (CRISPR)-administered genome editing is a powerful technique leading to a precisely targeted mutation in the genome of crop plants. Several CRISPR-Cas-derived editors that can execute precise genome alterations have been devised in addition to the indel mutations caused by the CRISPR-Cas nuclease. Allelic variants could also be produced, which serve as a potential genomic resource in crop variety development programs and developing resistance to plant viruses. Since the advent of next-generation sequencing (NGS), sufficient information on genome sequences and gene annotation of most crop plants has been available in the public domain. Such information could be best utilized to develop genome-edited crops with desired biotic and abiotic stress tolerance, nutritional quality, and higher yield. CRISPR can create null alleles by acting on exons or coding regions, and it can boost expression by acting on regulatory regions and ORFs. It can produce single or multiple mutations in homologous and non-homologous regions. Additionally, transgenes get eliminated in late generations due to segregation, leading to the development of transgene-free plants, which could be utilized without any regulatory issues in the near future.

Genome editing was used to successfully imitate tomato domestication, revealing the power of genome editing technologies (219). Several independent studies on CRISPR-engineered broad-spectrum disease resistance have demonstrated its versatile applications in various crop species. Moreover, several recent developments have been made to generate CRISPR-engineered dicotyledonous plants by *de novo* meristem induction (220), large DNA insertion (up to 2 kb) in rice (221), enhanced gene targeting efficiency through a heat-inducible CRISPR system in maize (222), and reconstructing the plant genome through genome engineering and somatic chromosome engineering, enabling genetic linkage (223). The CRISPR technique has been practically utilized to impart resistance to several plant RNA and DNA viruses. Geminiviruses are responsible for most economically important plant diseases, and to date, direct virus DNA targeting has been utilized to impart CRISPR-administered geminivirus resistance. However, this approach has limitations due to the eventual emergence of resistance-blocking strains and virus escape.

The off-target effect is the major challenge in CRISPR application, in which Cas9 nucleases cleave the wrong sites at the target genome. The SgRNA of 20 nucleotide sequences mainly controls Cas9 target specificity. Next to the target sequence, an adjacent PAM has the target specificity, but still, off-target cleavage occurs with 3-5 base pair mismatches. Additionally, the transformation of CRISPR-Cas-edited plants as a regeneration process is time-consuming, labor-intensive, and genotype-dependent. It could also cause somatic mutations during the process of regeneration. Low innate HDR efficiency is also a significant hurdle in CRISPR-based applications and causes unintended gene replacement and deletions. New developments for improving HDR efficiency could excel the applications of the CRISPR-Cas technique in crop plants. The future of CRISPR-Cas editing will lie in its simplified and robust use for the simultaneous targeting of multiple genetic loci in the same plant species, called multiplexing. This approach is emerging to the forefront in developing broad-spectrum and durable plant resistance against different viruses. These approaches, coupled with base and prime editor-based high-resolution modifications, are expected to make the target of zero hunger a reality in the coming years.

The current legal framework of genome-edited crops still regulates CRISPR-engineered crops as a process or product-based. Since regulatory guidelines vary from country to country, many countries consider genome-edited crops under strict GMO regulatory guidelines. Variety development takes a long time and costs more money because of these regulatory frameworks. Therefore, scientists and policymakers must collaborate to develop comprehensive plans for the integration of CRISPR-Cas edited crops to achieve food and nutritional security.

## Conclusions

Usually, crop losses are considered only in terms of reduction in yield, but it is equally important that reduction in the market value of crops due to the reduced quality and nutritional contents are taken into account. Understanding the concept and mechanism of plant-pathogen interactions and evolution, and additional analyses are essential to generate a holistic view of the combined effects of these factors on food and nutrition security worldwide. Using new emerging techniques and technologies like genome editing tools (CRISPR-Cas system), we can cope with some emerging issues on food production and increase nutritional quality in a short span of time in a more robust way. The current coronavirus disease-2019 (COVID-19) pandemic, in addition to the currently visible effects, might also put a negative effect on food security and nutrition in the long run. Thus, potential futuristic applications of the CRISPR tool will be to tackle the emerging and re-emerging viruses through targeted gene editing (singleplex and multiplex) to impart resistance to dreadful viral infections while simultaneously enhancing the yield and quality of end products. The scope of the application of CRISPR technologies and their rapidly evolving field will make global food and nutritional security realistic in years to come.

## Author Contributions

SKS, OPG, NP, AM, DS, and PS conceived the idea and designed the outline. SKS, NP, DS, AM, PS, JS, and SGK drafted the manuscript. AM, JS, PS, and DS prepared the illustrations. SKS, OPG, SK, and BB reviewed and improved the draft. All authors collected, compiled, analyzed, and interpreted the literature, contributed significantly to the article, and approved the submitted version.

## Conflict of Interest

The authors declare that the research was conducted in the absence of any commercial or financial relationships that could be construed as a potential conflict of interest.

## Publisher's Note

All claims expressed in this article are solely those of the authors and do not necessarily represent those of their affiliated organizations, or those of the publisher, the editors and the reviewers. Any product that may be evaluated in this article, or claim that may be made by its manufacturer, is not guaranteed or endorsed by the publisher.

## References

[1] United Nations. World Population Clock: 7.9 Billion People. (2021) - Worldometer. (2021). Available online at: https://www.worldometers.info/world-population/ (accessed July 28, 2021).

[2] FAO. The State of Food Security and Nutrition in the World 2020 | FAO | Food and Agriculture Organization of the United Nations. Food Agric Organ United Nations. (2020). Available online at: http://www.fao.org/publications/sofi/2021/en/ (accessed July 28, 2021).

[3] CABI.org. Global Burden of Crop Loss. Available online at: https://www.cabi.org/projects/global-burden-of-crop-loss/ (accessed July 29, 2021).

[4] Sastry KS. Impact of virus and viroid diseases on crop yields. In: Sastry KS editor. Plant Virus and Viroid Diseases in the Tropics. Dordrecht: Springer (2013). p. 99–159. 10.1007/978-94-007-6524-5_3

[5] Mumford RA Macarthur R Boonham N. The role and challenges of new diagnostic technology in plant biosecurity. Food Secur. (2016) 8:103–9. 10.1007/s12571-015-0533-y

[6] Andolfo G Iovieno P Frusciante L Ercolano MR. Genome-editing technologies for enhancing plant disease resistance. Front Plant Sci. (2016) 7:1813. 10.3389/fpls.2016.01813 27990151 PMC5130979

[7] de Ronde D Butterbach P Kormelink R. Dominant resistance against plant viruses. Front Plant Sci. (2014) 5:307. 10.3389/fpls.2014.00307 25018765 PMC4073217

[8] Andersen EJ Ali S Byamukama E Yen Y Nepal MP. Disease resistance mechanisms in plants. Genes. (2018) 9:339. 10.3390/genes9070339 29973557 PMC6071103

[9] Jones JDG Dangl JL. The plant immune system. Nature. (2006) 444:323–29. 10.1038/nature05286 17108957

[10] Simon-Loriere E Holmes EC. Why do RNA viruses recombine? Nat Rev Microbiol. (2011) 9:617–26. 10.1038/nrmicro2614 21725337 PMC3324781

[11] Jones RAC. Global plant virus disease pandemics and epidemics. Plants. (2021) 10:1–41. 10.3390/plants10020233 33504044 PMC7911862

[12] Sime SS Menkir A Adetimirin VO Gedil M Kumar PL. Validation of diagnostic markers for streak virus disease resistance in Maize. Agric. (2021) 11:1–11. 10.3390/agriculture11020130

[13] Bosque-Pérez NA. Eight decades of maize streak virus research. Virus Res. (2000) 71:107–21. 10.1016/S0168-1702(00)00192-1 11137166

[14] Burrows M Franc G Rush C Blunt T Ito D Kinzer K. Occurrence of viruses in wheat in the great plains region, 2008. Plant Heal Prog. (2009) 10:14. 10.1094/PHP-2009-0706-01-RS30812563

[15] Mishra GP Dikshit HK SVR Tripathi K Kumar RR Aski M Singh A Roy A Priti Kumari N. Yellow Mosaic Disease (YMD) of mungbean (Vigna radiata (L.) Wilczek): current status and management opportunities. Front Plant Sci. (2020) 11:918. 10.3389/fpls.2020.00918 32670329 PMC7327115

[16] Patil BL Kumar PL. Pigeonpea sterility mosaic virus: a legume-infecting Emaravirus from South Asia. Mol Plant Pathol. (2015) 16:775–86. 10.1111/mpp.12238 25640756 PMC6638375

[17] Mehboob-Ur-Rahman Khan AQ Rahmat Z Iqbal MA Zafar Y. Genetics and genomics of cotton leaf curl disease, its viral causal agents and whitefly vector: a way forward to sustain cotton fiber security. Front Plant Sci. (2017) 8:1157. 10.3389/fpls.2017.01157 28725230 PMC5495822

[18] Briddon RW Markham PG. Cotton leaf curl virus disease. Virus Res. (2000) 71:151–9. 10.1016/S0168-1702(00)00195-7 11137169

[19] Rajagopalan PA Naik A Katturi P Kurulekar M Kankanallu RS Anandalakshmi R. Dominance of resistance-breaking cotton leaf curl Burewala virus (CLCuBuV) in northwestern India. Arch Virol. (2012) 157:855–68. 10.1007/s00705-012-1225-y 22307170

[20] Moreno P Ambrós S Albiach-Martí MR Guerri J Peña L. Citrus tristeza virus: a pathogen that changed the course of the citrus industry. Mol Plant Pathol. (2008) 9:251–68. 10.1111/j.1364-3703.2007.00455.x 18705856 PMC6640355

[21] Sidharthan VK Sevanthi AM Jaiswal S Baranwal VK. Robust virome profiling and whole genome reconstruction of viruses and viroids enabled by use of available mRNA and sRNA-Seq datasets in grapevine (Vitis vinifera L.). Front Microbiol. (2020) 11:1232. 10.3389/fmicb.2020.01232 32582126 PMC7289960

[22] Fall ML Xu D Lemoyne P Ben Moussa IE Beaulieu C Carisse O. A diverse virome of leafroll-infected grapevine unveiled by dsRNA sequencing. Viruses. (2020) 12:1142–61. 10.20944/preprints202009.0646.v2 33050079 PMC7599845

[23] Rai R Sharma SK Kumar PV Baranwal VK. Evidence of novel genetic variants of Grapevine rupestris stem pitting-associated virus and intra-host diversity in Indian grapevine cultivars. Trop Plant Pathol. (2021) 46:576–80. 10.1007/s40858-021-00450-4

[24] James AP Geijskes RJ Dale JL Harding RM. Molecular characterisation of six badnavirus species associated with leaf streak disease of banana in East Africa. Ann Appl Biol. (2011) 158:346–53. 10.1111/j.1744-7348.2011.00466.x

[25] Sharma SK Vignesh Kumar P Geetanjali AS Pun KB Baranwal VK. Subpopulation level variation of banana streak viruses in India and common evolution of banana and sugarcane badnaviruses. Virus Genes. (2015) 50:450–65. 10.1007/s11262-015-1179-8 25672291

[26] Sharma SK Vignesh Kumar P Poswal R Rai R Swapna Geetanjali A Prabha K. Occurrence and distribution of banana streak disease and standardization of a reliable detection procedure for routine indexing of banana streak viruses in India. Sci Hortic. (2014) 179:277–83. 10.1016/j.scienta.2014.09.043

[27] Stainton D Martin DP Muhire BM Lolohea S Halafihi M Lepoint P. The global distribution of Banana bunchy top virus reveals little evidence for frequent recent, human-mediated long distance dispersal events. Virus Evol. (2015) 1:vev009. 10.1093/ve/vev009 27774281 PMC5014477

[28] Li C Yaegashi H Kishigami R Kawakubo A Yamagishi N Ito T. Apple russet ring and apple green crinkle diseases: fulfillment of Koch's Postulates by virome analysis, amplification of full-length cDNA of viral genomes, *in vitro* transcription of infectious viral RNAs, and reproduction of symptoms on fruits of apple T. Front Microbiol. (2020) 11:1627. 10.3389/fmicb.2020.01627 32754146 PMC7365870

[29] Wright AA Cross AR Harper SJ. A bushel of viruses: identification of seventeen novel putative viruses by RNA-seq in six apple trees. PLoS One. (2020) 15:e0227669. 10.1371/journal.pone.0227669 31929569 PMC6957168

[30] Nabi SU Baranwal VK. First report of apple hammerhead viroid infecting apple cultivars in India. Plant Dis. (2020) 104:3086. 10.1094/PDIS-12-19-2731-PDN30812563

[31] Nabi SU Baranwal VK Yadav MK Rao GP. Association of Apple necrotic mosaic virus (ApNMV) with mosaic disease in commercially grown cultivars of apple (Malus domestica Borkh) in India. 3 Biotech. (2020) 10:122. 10.1007/s13205-020-2117-6 32123646 PMC7026317

[32] Hajizadeh M Gibbs AJ Amirnia F Glasa M. The global phylogeny of Plum pox virus is emerging. J Gen Virol. (2019) 100:1457–68. 10.1099/jgv.0.001308 31418674

[33] Isogai M Yoshida T Nakanowatari C Yoshikawa N. Penetration of pollen tubes with accumulated Raspberry bushy dwarf virus into stigmas is involved in initial infection of maternal tissue and horizontal transmission. Virology. (2014) 452–453:247–53. 10.1016/j.virol.2014.02.001 24606702

[34] Thomas-Sharma S Wells-Hansen L Page R Kartanos V Saalau-Rojas E Lockhart BEL. Characterization of blueberry shock virus, an emerging ilarvirus in cranberry. Plant Dis. (2018) 102:91–7. 10.1094/PDIS-04-17-0551-RE 30673450

[35] Moriones E Navas-Castillo J. Tomato yellow leaf curl virus, an emerging virus complex causing epidemics worldwide. Virus Res. (2000) 71:123–34. 10.1016/S0168-1702(00)00193-3 11137167

[36] Oliver JE Whitfield AE. The genus tospovirus: emerging bunyaviruses that threaten food security. Annu Rev Virol. (2016) 3:101–24. 10.1146/annurev-virology-100114-055036 27578436

[37] Bananej K Desbiez C Girard M Wipf-Scheibel C Vahdat I Kheyr-Pour A. First report of cucumber vein yellowing virus on cucumber, melon, and watermelon in Iran. Plant Dis. (2006) 90:1113. 10.1094/PD-90-1113C 30781331

[38] Chikoti PC Mulenga RM Tembo M Sseruwagi P. Cassava mosaic disease: a review of a threat to cassava production in Zambia. J Plant Pathol. (2019) 101:467–77. 10.1007/s42161-019-00255-0 31983872 PMC6951474

[39] Flor HH. The complementary genic systems in flax and flax rust. Adv Genet. (1956) 8:29–54. 10.1016/S0065-2660(08)60498-8

[40] Flor HH. Current status of the gene-for-gene concept. Annu Rev Phytopathol. (1971) 9:275–96. 10.1146/annurev.py.09.090171.001423

[41] Lin S-S Henriques R Wu H-W Niu Q-W Yeh S-D Chua N-H. Strategies and mechanisms of plant virus resistance. Plant Biotechnol Rep. (2007) 1:125–34. 10.1007/s11816-007-0021-8

[42] Revers F Nicaise V. Plant resistance to infection by viruses. In: Wiley-Blackwell editor. Encyclopedia of Life Sciences. Chichester: John Wiley & Sons Ltd (2014). 10.1002/9780470015902.a0000757.pub3

[43] Dangwal M Mathad SM Patil BL. Novel strategies for engineering resistance to plant viral diseases. In: Prasad R Gill SS Tuteja N editors. New and Future Developments in Microbial Biotechnology and Bioengineering: Crop Improvement Through Microbial Biotechnology. Amsterdam: Elsevier (2018). p. 145–74. 10.1016/B978-0-444-63987-5.00007-4

[44] Solomon-Blackburn RM Barker H. Breeding virus resistant potatoes (Solanum tuberosum): a review of traditional and molecular approaches. Heredity. (2001) 86:17–35. 10.1046/j.1365-2540.2001.00799.x 11298812

[45] Ehlenfeldt MK Hanneman RE. The use of Endosperm Balance Number and 2n gametes to transfer exotic germplasm in potato. Theor Appl Genet. (1984) 68:155–61. 10.1007/BF00252332 24258959

[46] Johnston SA Hanneman RE. Manipulations of endosperm balance number overcome crossing barriers between diploid Solanum species. Science. (1982) 217:446–8. 10.1126/science.217.4558.446 17782980

[47] Ahloowalia BS Maluszynski M Nichterlein K. Global impact of mutation-derived varieties. Euphytica. (2004) 135:187–204. 10.1023/B:EUPH.0000014914.85465.4f

[48] FAO/IAEA. Mutant Varieties Database (2019). Available online at: https://www.iaea.org/resources/databases/mutant-varieties-database (accessed November 12, 2021).

[49] Fitch MMM Lehrer AT Komor E Moore PH. Elimination of Sugarcane yellow leaf virus from infected sugarcane plants by meristem tip culture visualized by tissue blot immunoassay. Plant Pathol. (2001) 50:676–80. 10.1046/j.1365-3059.2001.00639.x

[50] Singh MK Chandel V Hallan V Ram R Zaidi AA. Occurrence of Peanut stripe virus on patchouli and raising of virus-free patchouli plants by meristem tip culture. J Plant Dis Prot. (2009) 116:2–6. 10.1007/BF03356278

[51] Sasi S Bhat AI. In vitro elimination of Piper yellow mottle virus from infected black pepper through somatic embryogenesis and meristem-tip culture. Crop Prot. (2018) 103:39–45. 10.1016/j.cropro.2017.09.004

[52] Leu LS. Apical meristem culture and redifferentiation of callus masses to free some sugarcane systemic disease. Plant Prot Bull. (1978) 20:77–82.

[53] Austin S Baer MA Helgeson JP. Transfer of resistance to potato leaf roll virus from Solanum brevidens into Solanum tuberosum by somatic fusion. Plant Sci. (1985) 39:75–81. 10.1016/0168-9452(85)90195-5

[54] Gibson RW Jones MGK Fish N. Resistance to potato leaf roll virus and potato virus Y in somatic hybrids between dihaploid Solanum tuberosum and S. brevidens. Theor Appl Genet. (1988) 76:113–7. 10.1007/BF00288840 24231991

[55] Valkonen JPT Xu Y-S Pulli S Pehu E Rokka V-M. Transfer of resistance to potato leafroll virus, potato virus Y and potato virus X from Solarium brevidens to S. *tuberosum* through symmetric and designed asymmetric somatic hybridisation. Ann Appl Biol. (1994) 124:351–62. 10.1111/j.1744-7348.1994.tb04139.x

[56] Shi A Chen P Li D Zheng C Zhang B Hou A. Pyramiding multiple genes for resistance to soybean mosaic virus in soybean using molecular markers. Mol Breed. (2009) 23:113–24. 10.1007/s11032-008-9219-x

[57] Kumar Joshi R Nayak S. Gene pyramiding-A broad spectrum technique for developing durable stress resistance in crops. Biotechnol Mol Biol Rev. (2010) 5:51–60. 10.5897/BMBR2010.0006

[58] Werner K Friedt W Ordon F. Strategies for pyramiding resistance genes against the Barley Yellow Mosaic Virus complex (BaMMV, BaYMV, BaYMV-2). Mol Breed. (2005) 16:45–55. 10.1007/s11032-005-3445-2

[59] Abel PP Nelson RS De B Hoffmann N Rogers SG Fraley RT. Delay of disease development in transgenic plants that express the tobacco mosaic virus coat protein gene. Science. (1986) 232:738–43. 10.1126/science.3457472 3457472

[60] Ravelonandro M Scorza R Callahan A Levy L Jacquet C Monsion M. The use of transgenic fruit trees as a resistance strategy for virus epidemics: The plum pox (sharka) model. Virus Res. (2000) 71:63–9. 10.1016/S0168-1702(00)00188-X 11137162

[61] Bau HJ Cheng YH Yu TA Yang JS Yeh SD. Broad-spectrum resistance to different geographic strains of Papaya ringspot virus in coat protein gene transgenic papaya. Phytopathology. (2003) 93:112–20. 10.1094/PHYTO.2003.93.1.112 18944164

[62] Shepherd DN Mangwende T Martin DP Bezuidenhout M Thomson JA Rybicki EP. Inhibition of maize streak virus (MSV) replication by transient and transgenic expression of MSV replication-associated protein mutants. J Gen Virol. (2007) 88:325–36. 10.1099/vir.0.82338-0 17170465

[63] Elayabalan S Kalaiponmani K Subramaniam S Selvarajan R Panchanathan R Muthuvelayoutham R. Development of Agrobacterium-mediated transformation of highly valued hill banana cultivar Virupakshi (AAB) for resistance to BBTV disease. World J Microbiol Biotechnol. (2013) 29:589–96. 10.1007/s11274-012-1214-z 23184576

[64] Fuentes A Ramos PL Fiallo E Callard D Sánchez Y Peral R. Intron-hairpin RNA derived from replication associated protein C1 gene confers immunity to tomato yellow leaf curl virus infection in transgenic tomato plants. Transgenic Res. (2006) 15:291–304. 10.1007/s11248-005-5238-0 16779645

[65] Yeh S-D. Evaluation of induced mutants of papaya ringspot virus for control by cross protection. Phytopathology. (1984) 74:1086. 10.1094/Phyto-74-108630812563

[66] Praveen S Ramesh SV Mangrauthia SK. Transgenic approaches to combat plant viruses occurring in India. In: Mandal B Rao GP Baranwal VK Jain RK editors. A Century of Plant Virology in India. Singapore: Springer (2017). p. 783–805. 10.1007/978-981-10-5672-7_31

[67] Anderson JM Palukaitis P Zaitlin M. A defective replicase gene induces resistance to cucumber mosaic virus in transgenic tobacco plants. Proc Natl Acad Sci USA. (1992) 89:8759–63. 10.1073/pnas.89.18.8759 1528890 PMC50000

[68] Goregaoker SP Eckhardt LG Culver JN. Tobacco mosaic virus replicase-mediated cross-protection: contributions of RNA and protein-derived mechanisms. Virology. (2000) 273:267–75. 10.1006/viro.2000.0430 10915597

[69] Shivaprasad PV Thillaichidambaram P Balaji V Veluthambi K. Expression of full-length and truncated Rep genes from Mungbean yellow mosaic virus-Vigna inhibits viral replication in transgenic tobacco. Virus Genes. (2006) 33:365–74. 10.1007/s11262-006-0077-5 16991009

[70] Tepfer M. Risk assessment of virus-resistant transgenic plants. Annu Rev Phytopathol. (2002) 40:467–91. 10.1146/annurev.phyto.40.120301.093728 12147768

[71] Sanford JC Johnston SA. The concept of parasite-derived resistance-Deriving resistance genes from the parasite's own genome. J Theor Biol. (1985) 113:395–405. 10.1016/S0022-5193(85)80234-4

[72] Bendahmane M Chen I Asurmendi S Bazzini AA Szecsi J Beachy RN. Coat protein-mediated resistance to TMV infection of Nicotiana tabacum involves multiple modes of interference by coat protein. Virology. (2007) 366:107–16. 10.1016/j.virol.2007.03.052 17499327 PMC2139911

[73] Gonsalves D. Coat protein transgenic papaya: “acquired” immunity for controlling papaya ringspot virus. Curr Top Microbiol Immunol. (2002) 266:73–83. 10.1007/978-3-662-04700-2_6 12014204

[74] Ciuffo M Finetti-Sialer MM Gallitelli D Turina M. First report in Italy of a resistance-breaking strain of Tomato spotted wilt virus infecting tomato cultivars carrying the Sw5 resistance gene. Plant Pathol. (2005) 54:564. 10.1111/j.1365-3059.2005.01203.x

[75] Cosson P Schurdi-Levraud V Le QH Sicard O Caballero M Roux F. The RTM resistance to potyviruses in Arabidopsis thaliana: Natural variation of the RTM genes and evidence for the implication of additional genes. PLoS One. (2012) 7:e39169. 10.1371/journal.pone.0039169 22723957 PMC3377653

[76] Stein N Perovic D Kumlehn J Pellio B Stracke S Streng S. The eukaryotic translation initiation factor 4E confers multiallelic recessive Bymovirus resistance in Hordeum vulgare (L.). Plant J. (2005) 42:912–922. 10.1111/j.1365-313X.2005.02424.x 15941403

[77] Hart JP Griffiths PD. A series of eIF4E alleles at the Bc-3 locus are associated with recessive resistance to Clover yellow vein virus in common bean. Theor Appl Genet. (2013) 126:2849–63. 10.1007/s00122-013-2176-8 23933781

[78] ISAAA.org. International Service for the Acquisition of Agri-biotech Applications (2017). Available online at: https://www.isaaa.org/ (accessed November 5, 2021).

[79] Dangl JL Horvath DM Staskawicz BJ. Pivoting the plant immune system from dissection to deployment. Science. (2013) 341:746–51. 10.1126/science.1236011 23950531 PMC3869199

[80] Ronald PC. Lab to farm: applying research on plant genetics and genomics to crop improvement. PLoS Biol. (2014) 12:1–6. 10.1371/journal.pbio.1001878 24915201 PMC4051633

[81] Waterhouse PM Wang MB Lough T. Gene silencing as an adaptive defence against viruses. Nature. (2001) 411:834–42. 10.1038/35081168 11459066

[82] Schwarz DS Hutvágner G Haley B Zamore PD. Evidence that siRNAs function as guides, not primers, in the Drosophila and human RNAi pathways. Mol Cell. (2002) 10:537–48. 10.1016/S1097-2765(02)00651-2 12408822

[83] Worrall EA Bravo-Cazar A Nilon AT Fletcher SJ Robinson KE Carr JP. Exogenous application of RNAi-inducing double-stranded RNA inhibits aphid-mediated transmission of a plant virus. Front Plant Sci. (2019) 10:265. 10.3389/fpls.2019.00265 30930914 PMC6429036

[84] Montes C Castro Á Barba P Rubio J Sánchez E Carvajal D. Differential RNAi responses of Nicotiana benthamiana individuals transformed with a hairpin-inducing construct during Plum pox virus challenge. Virus Genes. (2014) 49:325–38. 10.1007/s11262-014-1093-5 24964777

[85] Yang X Niu L Zhang W Yang J Xing G He H. RNAi-mediated SMV P3 cistron silencing confers significantly enhanced resistance to multiple Potyvirus strains and isolates in transgenic soybean. Plant Cell Rep. (2018) 37:103–14. 10.1007/s00299-017-2186-0 28756582

[86] Guo J Gao S Lin Q Wang H Que Y Xu L. Transgenic sugarcane resistant to sorghum mosaic virus based on coat protein gene silencing by RNA interference. Biomed Res Int. (2015) 2015:861907. 10.1155/2015/861907 25685813 PMC4317601

[87] Cruz ARR Aragão FJL. RNAi-based enhanced resistance to Cowpea severe mosaic virus and Cowpea aphid-borne mosaic virus in transgenic cowpea. Plant Pathol. (2014) 63:831–7. 10.1111/ppa.12178

[88] Chen L Ren Y Zhang Y Xu J Zhang Z Wang Y. Genome-wide profiling of novel and conserved Populus microRNAs involved in pathogen stress response by deep sequencing. Planta. (2012) 235:873–83. 10.1007/s00425-011-1548-z 22101925

[89] Du J Wu G Zhou Z Zhang J Li M Sun M. Identification of microRNAs regulated by tobacco curly shoot virus co-infection with its betasatellite in Nicotiana benthamiana. Virol J. (2019) 16:1–12. 10.1186/s12985-019-1234-5 31699111 PMC6836351

[90] Zhang C Ding Z Wu K Yang L Li Y Yang Z. Suppression of jasmonic acid-mediated defense by viral-inducible MicroRNA319 facilitates virus infection in rice. Mol Plant. (2016) 9:1302–14. 10.1016/j.molp.2016.06.014 27381440

[91] Romanel E Silva TF Corrêa RL Farinelli L Hawkins JS Schrago CEG. Global alteration of microRNAs and transposon-derived small RNAs in cotton (Gossypium hirsutum) during Cotton leafroll dwarf polerovirus (CLRDV) infection. Plant Mol Biol. (2012) 80:443–60. 10.1007/s11103-012-9959-1 22987114

[92] Kaldis A Berbati M Melita O Reppa C Holeva M Otten P. Exogenously applied dsRNA molecules deriving from the Zucchini yellow mosaic virus (ZYMV) genome move systemically and protect cucurbits against ZYMV. Mol Plant Pathol. (2018) 19:883–95. 10.1111/mpp.12572 28621835 PMC6638139

[93] Lau SE Mazumdar P Hee TW Song ALA Othman RY Harikrishna JA. Crude extracts of bacterially-expressed dsRNA protect orchid plants against *Cymbidium mosaic* virus during transplantation from *in vitro* culture. J Hortic Sci Biotechnol. (2014) 89:569–76. 10.1080/14620316.2014.11513122

[94] Namgial T Kaldis A Chakraborty S Voloudakis A. Topical application of double-stranded RNA molecules containing sequences of Tomato leaf curl virus and *Cucumber mosaic* virus confers protection against the cognate viruses. Physiol Mol Plant Pathol. (2019) 108:101432. 10.1016/j.pmpp.2019.101432

[95] Li H Yang Y Hong W Huang M Wu M Zhao X. Applications of genome editing technology in the targeted therapy of human diseases: mechanisms, advances and prospects. Signal Transduct Target Ther. (2020) 5:1–23. 10.1038/s41392-019-0089-y 32296011 PMC6946647

[96] Yin K Qiu JL. Genome editing for plant disease resistance: applications and perspectives. Philos Trans R Soc B Biol Sci. (2019) 374:20180322. 10.1098/rstb.2018.0322 30967029 PMC6367152

[97] Wimmer F Beisel CL. CRISPR-Cas systems and the paradox of self-targeting spacers. Front Microbiol. (2020) 10:3078. 10.3389/fmicb.2019.03078 32038537 PMC6990116

[98] Ishino Y Shinagawa H Makino K Amemura M Nakatura A. Nucleotide sequence of the iap gene, responsible for alkaline phosphatase isoenzyme conversion in *Escherichia coli*, and identification of the gene product. J Bacteriol. (1987) 169:5429–5433. 10.1128/jb.169.12.5429-5433.1987 3316184 PMC213968

[99] Jansen R Van Embden JDA Gaastra W Schouls LM. Identification of genes that are associated with DNA repeats in prokaryotes. Mol Microbiol. (2002) 43:1565–75. 10.1046/j.1365-2958.2002.02839.x 11952905

[100] Lander ES. The heroes of CRISPR. Cell. (2016) 164:18–28. 10.1016/j.cell.2015.12.041 26771483

[101] Mojica FJM Díez-Villaseñor C García-Martínez J Soria E. Intervening sequences of regularly spaced prokaryotic repeats derive from foreign genetic elements. J Mol Evol. (2005) 60:174–82. 10.1007/s00239-004-0046-3 15791728

[102] Lusser M Parisi C Plan D Rodríguez-Cerezo E. Deployment of new biotechnologies in plant breeding. Nat Biotechnol. (2012) 30:231–9. 10.1038/nbt.2142 22398616 PMC7097357

[103] Zhu C Bortesi L Baysal C Twyman RM Fischer R Capell T. Characteristics of genome editing mutations in cereal crops. Trends Plant Sci. (2017) 22:38–52. 10.1016/j.tplants.2016.08.009 27645899

[104] Feng Z Mao Y Xu N Zhang B Wei P Yang DL. Multigeneration analysis reveals the inheritance, specificity, and patterns of CRISPR/Cas-induced gene modifications in Arabidopsis. Proc Natl Acad Sci USA. (2014) 111:4632–7. 10.1073/pnas.1400822111 24550464 PMC3970504

[105] Ishizaki T. CRISPR/Cas9 in rice can induce new mutations in later generations, leading to chimerism and unpredicted segregation of the targeted mutation. Mol Breed. (2016) 36:165–80. 10.1007/s11032-016-0591-7

[106] Zong Y Wang Y Li C Zhang R Chen K Ran Y. Precise base editing in rice, wheat and maize with a Cas9-cytidine deaminase fusion. Nat Biotechnol. (2017) 35:438–40. 10.1038/nbt.3811 28244994

[107] Koonin E V. Makarova KS. Origins and evolution of CRISPR-Cas systems. Philos Trans R Soc B Biol Sci. (2019) 374:20180087. 10.1098/rstb.2018.0087 30905284 PMC6452270

[108] Rath D Amlinger L Rath A Lundgren M. The CRISPR-Cas immune system: biology, mechanisms and applications. Biochimie. (2015) 117:119–28. 10.1016/j.biochi.2015.03.025 25868999

[109] Pourcel C Salvignol G Vergnaud G. CRISPR elements in Yersinia pestis acquire new repeats by preferential uptake of bacteriophage DNA, and provide additional tools for evolutionary studies. Microbiology. (2005) 151:653–63. 10.1099/mic.0.27437-0 15758212

[110] Song G Jia M Chen K Kong X Khattak B Xie C. CRISPR/Cas9: a powerful tool for crop genome editing. Crop J. (2016) 4:75–82. 10.1016/j.cj.2015.12.002

[111] Lieber MR. The mechanism of double-strand DNA break repair by the nonhomologous DNA end-joining pathway. Annu Rev Biochem. (2010) 79:181–211. 10.1146/annurev.biochem.052308.093131 20192759 PMC3079308

[112] Ren C Liu X Zhang Z Wang Y Duan W Li S. CRISPR/Cas9-mediated efficient targeted mutagenesis in Chardonnay (Vitis vinifera L.). Sci Rep. (2016) 6:32289. 10.1038/srep32289 27576893 PMC5006071

[113] Cong L Ran FA Cox D Lin S Barretto R Habib N. Multiplex genome engineering using CRISPR/Cas systems. Science. (2013) 339:819–23. 10.1126/science.1231143 23287718 PMC3795411

[114] Kim D Kang BC Kim JS. Identifying genome-wide off-target sites of CRISPR RNA–guided nucleases and deaminases with Digenome-seq. Nat Protoc. (2021) 16:1170–92. 10.1038/s41596-020-00453-6 33462439

[115] Rees HA Liu DR. Base editing: precision chemistry on the genome and transcriptome of living cells. Nat Rev Genet. (2018) 19:770–88. 10.1038/s41576-018-0059-1 30323312 PMC6535181

[116] Karvelis T Bigelyte G Young JK Hou Z Zedaveinyte R Pociute K. PAM recognition by miniature CRISPR-Cas14 triggers programmable double-stranded DNA cleavage. Nucleic Acids Res. (2020) 48:5016–23. 10.1093/nar/gkaa208 32246713 PMC7229846

[117] Collias D Beisel CL. CRISPR technologies and the search for the PAM-free nuclease. Nat Commun. (2021) 12:555. 10.1038/s41467-020-20633-y 33483498 PMC7822910

[118] Molla KA Sretenovic S Bansal KC Qi Y. Precise plant genome editing using base editors and prime editors. Nat Plants. (2021) 7:1166–87. 10.1038/s41477-021-00991-1 34518669

[119] Yeh WH Chiang H Rees HA Edge ASB Liu DR. *In vivo* base editing of post-mitotic sensory cells. Nat Commun. (2018) 9:2184. 10.1038/s41467-018-04580-3 29872041 PMC5988727

[120] Ahmad S Wei X Sheng Z Hu P Tang S. CRISPR/Cas9 for development of disease resistance in plants: recent progress, limitations and future prospects. Brief Funct Genomics. (2018) 19:26–39. 10.1093/bfgp/elz041 31915817

[121] Thomas HR Percival SM Yoder BK Parant JM. High-throughput genome editing and phenotyping facilitated by high resolution melting curve analysis. PLoS ONE. (2014) 9:e114632. 10.1371/journal.pone.0114632 25503746 PMC4263700

[122] Vouillot L Thélie A Pollet N. Comparison of T7E1 and surveyor mismatch cleavage assays to detect mutations triggered by engineered nucleases. G3 Genes, Genomes, Genet. (2015) 5:407–15. 10.1534/g3.114.015834 25566793 PMC4349094

[123] Zischewski J Fischer R Bortesi L. Detection of on-target and off-target mutations generated by CRISPR/Cas9 and other sequence-specific nucleases. Biotechnol Adv. (2017) 35:95–104. 10.1016/j.biotechadv.2016.12.003 28011075

[124] Zhu X Xu Y Yu S Lu L Ding M Cheng J. An efficient genotyping method for genome-modified animals and human cells generated with CRISPR/Cas9 system. Sci Rep. (2014) 4:6420. 10.1038/srep06420 25236476 PMC4168274

[125] Huang MC Cheong WC Lim LS Li MH. A simple, high sensitivity mutation screening using Ampligase mediated T7 endonuclease I and Surveyor nuclease with microfluidic capillary electrophoresis. Electrophoresis. (2012) 33:788–96. 10.1002/elps.201100460 22437793

[126] Harayama T Riezman H. Detection of genome-edited mutant clones by a simple competition-based PCR method. PLoS One. (2017) 12:e0179165. 10.1371/journal.pone.0179165 28586390 PMC5460891

[127] Ota S Hisano Y Muraki M Hoshijima K Dahlem TJ Grunwald DJ. Efficient identification of TALEN-mediated genome modifications using heteroduplex mobility assays. Genes Cells. (2013) 18:450–8. 10.1111/gtc.12050 23573916 PMC4834911

[128] Biswas S Li R Hong J Zhao X Yuan Z Zhang D. Effective identification of CRISPR/Cas9-induced and naturally occurred mutations in rice using a multiplex ligation-dependent probe amplification-based method. Theor Appl Genet. (2020) 133:2323–34. 10.1007/s00122-020-03600-5 32405769

[129] Peng C Wang H Xu X Wang X Chen X Wei W. High-throughput detection and screening of plants modified by gene editing using quantitative real-time polymerase chain reaction. Plant J. (2018) 95:557–67. 10.1111/tpj.13961 29761864

[130] Veres A Gosis BS Ding Q Collins R Ragavendran A Brand H. Low incidence of Off-target mutations in individual CRISPR-Cas9 and TALEN targeted human stem cell clones detected by whole-genome sequencing. Cell Stem Cell. (2014) 15:27–30. 10.1016/j.stem.2014.04.020 24996167 PMC4082799

[131] Kim JM Kim D Kim S Kim JS. Genotyping with CRISPR-Cas-derived RNA-guided endonucleases. Nat Commun. (2014) 5:3157. 10.1038/ncomms4157 24445736

[132] Yu C Zhang Y Yao S Wei Y. A PCR based protocol for detecting indel mutations induced by TALENs and CRISPR/Cas9 in zebrafish. PLoS One. (2014) 9:e98282. 10.1371/journal.pone.0098282 24901507 PMC4046980

[133] Brinkman EK Chen T Amendola M Van Steensel B. Easy quantitative assessment of genome editing by sequence trace decomposition. Nucleic Acids Res. (2014) 42:e168. 10.1093/nar/gku936 25300484 PMC4267669

[134] Güell M Yang L Church GM. Genome editing assessment using CRISPR Genome Analyzer (CRISPR-GA). Bioinformatics. (2014) 30:2968–70. 10.1093/bioinformatics/btu427 24990609 PMC4184265

[135] Hua Y Wang C Huang J Wang K. A simple and efficient method for CRISPR/Cas9-induced mutant screening. J Genet Genomics. (2017) 44:207–13. 10.1016/j.jgg.2017.03.005 28416245

[136] Lonowski LA Narimatsu Y Riaz A Delay CE Yang Z Niola F. Genome editing using FACS enrichment of nuclease-expressing cells and indel detection by amplicon analysis. Nat Protoc. (2017) 12:581–603. 10.1038/nprot.2016.165 28207001 PMC7250141

[137] Kohata R Koitabashi K Kitashiba H Nishio T. Sensitive mutant detection by concentrating mutant DNA with allele-specific capture and its application to analysis of contaminated grains in rice. Plant Cell Rep. (2018) 37:865–72. 10.1007/s00299-018-2274-9 29532250

[138] Guo J Li K Jin L Xu R Miao K Yang F. A simple and cost-effective method for screening of CRISPR/Cas9-induced homozygous/biallelic mutants. Plant Methods. (2018) 14:40. 10.1186/s13007-018-0305-8 29872452 PMC5972395

[139] Zhang H Zhang J Wei P Zhang B Gou F Feng Z. The CRISPR/Cas9 system produces specific and homozygous targeted gene editing in rice in one generation. Plant Biotechnol J. (2014) 12:797–807. 10.1111/pbi.12200 24854982

[140] Barakate A Stephens J. An overview of crispr-based tools and their improvements: New opportunities in understanding plant-pathogen interactions for better crop protection. Front Plant Sci. (2016) 7:765. 10.3389/fpls.2016.00765 27313592 PMC4887484

[141] Kalinina NO Khromov A Love AJ Taliansky ME. CRISPR applications in plant virology: virus resistance and beyond. Phytopathology. (2020) 110:18–28. 10.1094/PHYTO-07-19-0267-IA 31433273

[142] Yin K Han T Xie K Zhao J Song J Liu Y. Engineer complete resistance to cotton leaf curl Multan virus by the CRISPR/Cas9 system in *Nicotiana benthamiana*. Phytopathol Res. (2019) 1:1–9. 10.1186/s42483-019-0017-7

[143] Tripathi JN Ntui VO Ron M Muiruri SK Britt A Tripathi L. CRISPR/Cas9 editing of endogenous banana streak virus in the B genome of Musa spp. overcomes a major challenge in banana breeding. Commun Biol. (2019) 2:46. 10.1038/s42003-019-0288-7 30729184 PMC6355771

[144] Kis A Hamar É Tholt G Bán R Havelda Z. Creating highly efficient resistance against wheat dwarf virus in barley by employing CRISPR/Cas9 system. Plant Biotechnol J. (2019) 17:1004–06. 10.1111/pbi.13077 30633425 PMC6523583

[145] Ali Z Abulfaraj A Idris A Ali S Tashkandi M Mahfouz MM. CRISPR/Cas9-mediated viral interference in plants. Genome Biol. (2015) 16:238. 10.1186/s13059-015-0799-6 26556628 PMC4641396

[146] Baltes NJ Hummel AW Konecna E Cegan R Bruns AN Bisaro DM. Conferring resistance to geminiviruses with the CRISPR-Cas prokaryotic immune system. Nat Plants. (2015) 1:15145. 10.1038/nplants.2015.145 34824864 PMC8612103

[147] Ji X Zhang H Zhang Y Wang Y Gao C. Establishing a CRISPR–Cas-like immune system conferring DNA virus resistance in plants. Nat Plants. (2015) 1:15144. 10.1038/nplants.2015.144 27251395

[148] Ali Z Ali S Tashkandi M Zaidi SSEA Mahfouz MM. CRISPR/Cas9-mediated immunity to geminiviruses: differential interference and evasion. Sci Rep. (2016) 6:26912. 10.1038/srep26912 27225592 PMC4881029

[149] Tashkandi M Ali Z Aljedaani F Shami A Mahfouz MM. Engineering resistance against Tomato yellow leaf curl virus via the CRISPR/Cas9 system in tomato. Plant Signal Behav. (2018) 13:e1525996. 10.1101/237735 30289378 PMC6204811

[150] Zhang T Zheng Q Yi X An H Zhao Y Ma S. Establishing RNA virus resistance in plants by harnessing CRISPR immune system. Plant Biotechnol J. (2018) 16:1415–23. 10.1111/pbi.12881 29327438 PMC6041442

[151] Aman R Ali Z Butt H Mahas A Aljedaani F Khan MZ. RNA virus interference via CRISPR/Cas13a system in plants. Genome Biol. (2018) 19:1–9. 10.1186/s13059-017-1381-1 29301551 PMC5755456

[152] Zhang T Zhao Y Ye J Cao X Xu C Chen B. Establishing CRISPR/Cas13a immune system conferring RNA virus resistance in both dicot and monocot plants. Plant Biotechnol J. (2019) 17:1185–7. 10.1111/pbi.13095 30785668 PMC6576088

[153] Zhan X Zhang F Zhong Z Chen R Wang Y Chang L. Generation of virus-resistant potato plants by RNA genome targeting. Plant Biotechnol J. (2019) 17:1814–22. 10.1111/pbi.13102 30803101 PMC6686122

[154] Wang T Deng Z Zhang X Wang H Wang Y Liu X. Tomato DCL2b is required for the biosynthesis of 22-nt small RNAs, the resulting secondary siRNAs, and the host defense against ToMV. Hortic Res. (2018) 5:1–14. 10.1038/s41438-018-0073-7 30181890 PMC6119189

[155] Wang Z Hardcastle TJ Pastor AC Yip WH Tang S Baulcombe DC. A novel DCL2-dependent miRNA pathway in tomato affects susceptibility to RNA viruses. Genes Dev. (2018) 32:1155–60. 10.1101/gad.313601.118 30150254 PMC6120711

[156] Chandrasekaran J Brumin M Wolf D Leibman D Klap C Pearlsman M. Development of broad virus resistance in non-transgenic cucumber using CRISPR/Cas9 technology. Mol Plant Pathol. (2016) 17:1140–53. 10.1111/mpp.12375 26808139 PMC6638350

[157] Pyott DE Sheehan E Molnar A. Engineering of CRISPR/Cas9-mediated potyvirus resistance in transgene-free Arabidopsis plants. Mol Plant Pathol. (2016) 17:1276–88. 10.1111/mpp.12417 27103354 PMC5026172

[158] Gomez MA Lin ZD Moll T Chauhan RD Hayden L Renninger K. Simultaneous CRISPR/Cas9-mediated editing of cassava eIF4E isoforms nCBP-1 and nCBP-2 reduces cassava brown streak disease symptom severity and incidence. Plant Biotechnol J. (2019) 17:421–34. 10.1111/pbi.12987 30019807 PMC6335076

[159] Bastet A Zafirov D Giovinazzo N Guyon-Debast A Nogué F Robaglia C. Mimicking natural polymorphism in eIF4E by CRISPR-Cas9 base editing is associated with resistance to potyviruses. Plant Biotechnol J. (2019) 17:1736–50. 10.1111/pbi.13096 30784179 PMC6686125

[160] Makhotenko A V. Khromov A V. Snigir EA Makarova SS Makarov V V. Suprunova TP. Functional analysis of coilin in virus resistance and stress tolerance of potato *Solanum tuberosum* using CRISPR-Cas9 Editing. Dokl Biochem Biophys. (2019) 484:88–91. 10.1134/S1607672919010241 31012023

[161] Mehta D Stürchler A Anjanappa RB Zaidi SSEA Hirsch-Hoffmann M Gruissem W. Linking CRISPR-Cas9 interference in cassava to the evolution of editing-resistant geminiviruses. Genome Biol. (2019) 20:80. 10.1186/s13059-019-1678-3 31018865 PMC6482539

[162] Zhang Y Qian L Wei W Wang Y Wang B Lin P. Paired design of dCas9 as a systematic platform for the detection of featured nucleic acid sequences in pathogenic strains. ACS Synth Biol. (2017) 6:211–6. 10.1021/acssynbio.6b00215 27718551

[163] Chen JS Ma E Harrington LB Da Costa M Tian X Palefsky JM. CRISPR-Cas12a target binding unleashes indiscriminate single-stranded DNase activity. Science. (2018) 360:436–9. 10.1126/science.aar6245 29449511 PMC6628903

[164] Gootenberg JS Abudayyeh OO Lee JW Essletzbichler P Dy AJ Joung J. Nucleic acid detection with CRISPR-Cas13a/C2c2. Science. (2017) 356:438–42. 10.1126/science.aam9321 28408723 PMC5526198

[165] Harrington LB Burstein D Chen JS Paez-Espino D Ma E Witte IP. Programmed DNA destruction by miniature CRISPR-Cas14 enzymes. Science. (2018) 362:839–42. 10.1126/science.aav4294 30337455 PMC6659742

[166] Li C Zong Y Wang Y Jin S Zhang D Song Q. Expanded base editing in rice and wheat using a Cas9-adenosine deaminase fusion. Genome Biol. (2018) 19:1–9. 10.1186/s13059-018-1443-z 29807545 PMC5972399

[167] Chaijarasphong T Thammachai T Itsathitphaisarn O Sritunyalucksana K Suebsing R. Potential application of CRISPR-Cas12a fluorescence assay coupled with rapid nucleic acid amplification for detection of white spot syndrome virus in shrimp. Aquaculture. (2019) 512:734340. 10.1016/j.aquaculture.2019.734340

[168] Myhrvold C Freije CA Gootenberg JS Abudayyeh OO Metsky HC Durbin AF. Field-deployable viral diagnostics using CRISPR-Cas13. Science. (2018) 360:444–8. 10.1126/science.aas8836 29700266 PMC6197056

[169] Zhang M Liu C Shi Y Wu J Wu J Chen H. Selective endpoint visualized detection of Vibrio parahaemolyticus with CRISPR/Cas12a assisted PCR using thermal cycler for on-site application. Talanta. (2020) 214:120818. 10.1016/j.talanta.2020.120818 32278427

[170] Zhang Y mu Zhang Y Xie K. Evaluation of CRISPR/Cas12a-based DNA detection for fast pathogen diagnosis and GMO test in rice. Mol Breed. (2020) 40:1–12. 10.1007/s11032-019-1092-2

[171] Mahas A Hassan N Aman R Marsic T Wang Q Ali Z. Lamp-coupled crispr–cas12a module for rapid and sensitive detection of plant dna viruses. Viruses. (2021) 13:466. 10.3390/v13030466 33808947 PMC8001329

[172] Ramachandran V Weiland JJ Bolton MD. CRISPR-based isothermal next-generation diagnostic method for virus detection in sugarbeet. Front Microbiol. (2021) 12:1760. 10.3389/fmicb.2021.679994 34305843 PMC8297705

[173] Alon DM Hak H Bornstein M Pines G Spiegelman Z. Differential detection of the tobamoviruses tomato mosaic virus (ToMV) and tomato brown rugose fruit virus (ToBRFV) using CRISPR-Cas12a. Plants. (2021) 10:1256. 10.3390/plants10061256 34205558 PMC8234260

[174] Khan S Mahmood MS Ur Rahman S Rizvi F Ahmad A. Evaluation of the CRISPR/Cas9 system for the development of resistance against Cotton leaf curl virus in model plants. Plant Prot Sci. (2020) 56:154–62. 10.17221/105/2019-PPS105

[175] Aman R Mahas A Marsic T Hassan N Mahfouz MM. Efficient, rapid, and sensitive detection of plant RNA viruses with One-Pot RT-RPA–CRISPR/Cas12a assay. Front Microbiol. (2020) 11:3277. 10.3389/fmicb.2020.610872 33391239 PMC7773598

[176] Tripathi L Ntui VO Tripathi JN Kumar PL. Application of CRISPR/Cas for diagnosis and management of viral diseases of banana. Front Microbiol. (2021) 11:3622. 10.3389/fmicb.2020.609784 33584573 PMC7873300

[177] Jiao J Kong K Han J Song S Bai T Song C. Field detection of multiple RNA viruses/viroids in apple using a CRISPR/Cas12a-based visual assay. Plant Biotechnol J. (2021) 19:394–405. 10.1111/pbi.13474 32886837 PMC7868969

[178] Mendes RJ Luz JP Santos C Tavares F. CRISPR genotyping as complementary tool for epidemiological surveillance of Erwinia amylovora outbreaks. PLoS One. (2021) 16:e0250280. 10.1371/journal.pone.0250280 33861806 PMC8051791

[179] Gootenberg JS Abudayyeh OO Kellner MJ Joung J Collins JJ Zhang F. Multiplexed and portable nucleic acid detection platform with Cas13, Cas12a and Csm6. Science. (2018) 360:439–44. 10.1126/science.aaq0179 29449508 PMC5961727

[180] Kaminski MM Abudayyeh OO Gootenberg JS Zhang F Collins JJ. CRISPR-based diagnostics. Nat Biomed Eng. (2021) 5:643–56. 10.1038/s41551-021-00760-7 34272525

[181] Shahzad R Jamil S Ahmad S Nisar A Khan S Amina Z. Biofortification of cereals and pulses using new breeding techniques: current and future perspectives. Front Nutr. (2021) 8:665. 10.3389/fnut.2021.721728 34692743 PMC8528959

[182] Tabassum J Ahmad S Hussain B Mawia AM Zeb A Ju L. Applications and potential of genome-editing systems in rice improvement: current and future perspectives. Agronomy. (2021) 11:1359. 10.3390/agronomy11071359

[183] Mao T Zhu M Sheng Z Shao G Jiao G Mawia AM. Effects of grain shape genes editing on appearance quality of erect-panicle geng/japonica rice. Rice. (2021) 14:1–7. 10.1186/s12284-021-00517-5 34374880 PMC8355294

[184] Monsur MB Shao G Lv Y Ahmad S Wei X Hu P. Base editing: the ever expanding clustered regularly interspaced short palindromic repeats (CRISPR) tool kit for precise genome editing in plants. Genes. (2020) 11:466. 10.3390/genes11040466 32344599 PMC7231171

[185] Abd-Elsalam KA Lim K-T. CRISPR and RNAi Systems Nanobiotechnology Approaches to Plant Breeding and Protection CRISPR and RNAi Systems Nanobiotechnology Approaches to Plant Breeding and Protection. Amsterdam: Elsevier (2021). 10.1016/B978-0-12-821910-2.00019-9

[186] Ahmad S Shahzad R Jamil S Nisar A Khan Z Kanwal S. CRISPR mediated genome editing for developing climate-resilient monocot and dicot crops. In: Aftab T Roychoudhury editors. Plant Perspectives to Global Climate Changes. London: Academic Press (2021). p. 393–411. 10.1016/B978-0-323-85665-2.00006-6

[187] Chen K Wang Y Zhang R Zhang H Gao C. CRISPR/Cas genome editing and precision plant breeding in agriculture. Annu Rev Plant Biol. (2019) 70:667–97. 10.1146/annurev-arplant-050718-100049 30835493

[188] Tiwari M Kumar Trivedi P Pandey A. Emerging tools and paradigm shift of gene editing in cereals, fruits, and horticultural crops for enhancing nutritional value and food security. Food Energy Secur. (2021) 10:e258. 10.1002/fes3.25825855820

[189] Li M Li X Zhou Z Wu P Fang M Pan X. Reassessment of the four yield-related genes Gn1a, DEP1, GS3, and IPA1 in rice using a CRISPR/Cas9 system. Front Plant Sci. (2016) 7:377. 10.3389/fpls.2016.00377 27066031 PMC4811884

[190] Li S Gao F Xie K Zeng X Cao Y Zeng J. The OsmiR396c-OsGRF4-OsGIF1 regulatory module determines grain size and yield in rice. Plant Biotechnol J. (2016) 14:2134–46. 10.1111/pbi.12569 27107174 PMC5095787

[191] Liu J Chen J Zheng X Wu F Lin Q Heng Y. GW5 acts in the brassinosteroid signalling pathway to regulate grain width and weight in rice. Nat Plants. (2017) 3:17043. 10.1038/nplants.2017.43 28394310

[192] Lu K Wu B Wang J Zhu W Nie H Qian J. Blocking amino acid transporter OsAAP3 improves grain yield by promoting outgrowth buds and increasing tiller number in rice. Plant Biotechnol J. (2018) 16:1710–22. 10.1111/pbi.12907 29479779 PMC6131477

[193] Zhang Y Li D Zhang D Zhao X Cao X Dong L. Analysis of the functions of TaGW2 homoeologs in wheat grain weight and protein content traits. Plant J. (2018) 94:857–66. 10.1111/tpj.13903 29570880

[194] Shi J Gao H Wang H Lafitte HR Archibald RL Yang M. ARGOS8 variants generated by CRISPR-Cas9 improve maize grain yield under field drought stress conditions. Plant Biotechnol J. (2017) 15:207–16. 10.1111/pbi.12603 27442592 PMC5258859

[195] Rodríguez-Leal D Lemmon ZH Man J Bartlett ME Lippman ZB. Engineering quantitative trait variation for crop improvement by genome editing. Cell. (2017) 171:470–80.e8. 10.1016/j.cell.2017.08.030 28919077

[196] Ahmad S Tang L Shahzad R Mawia AM Rao GS Jamil S. CRISPR-based crop improvements: a way forward to achieve zero hunger. J Agric Food Chem. (2021) 69:8307–23. 10.1021/acs.jafc.1c02653 34288688

[197] Fiaz S Ahmad S Ali Noor M Wang X Younas A Riaz A. Applications of the CRISPR/Cas9 system for rice grain quality improvement: perspectives and opportunities. Int J Mol Sci. (2019) 20:888. 10.3390/ijms20040888 30791357 PMC6412304

[198] Hui S Li H Mawia AM Zhou L Cai J Ahmad S. Production of aromatic three-line hybrid rice using novel alleles of BADH2. Plant Biotechnol J. (2021) 1–16. 10.1111/pbi.13695 34465003 PMC8710899

[199] Waltz E. With a free pass, CRISPR-edited plants reach market in record time. Nat Biotechnol. (2018) 36:6–7. 10.1038/nbt0118-6b 29319694

[200] Sun Y Jiao G Liu Z Zhang X Li J Guo X. Generation of high-amylose rice through CRISPR/Cas9-mediated targeted mutagenesis of starch branching enzymes. Front Plant Sci. (2017) 8:298. 10.3389/fpls.2017.00298 28326091 PMC5339335

[201] Shan Q Zhang Y Chen K Zhang K Gao C. Creation of fragrant rice by targeted knockout of the OsBADH2 gene using TALEN technology. Plant Biotechnol J. (2015) 13:791–800. 10.1111/pbi.12312 25599829

[202] Tang L Mao B Li Y Lv Q Zhang L Chen C. Knockout of OsNramp5 using the CRISPR/Cas9 system produces low Cd-accumulating indica rice without compromising yield. Sci Rep. (2017) 7:14438. 10.1038/s41598-017-14832-9 29089547 PMC5663754

[203] Abe K Araki E Suzuki Y Toki S Saika H. Production of high oleic/low linoleic rice by genome editing. Plant Physiol Biochem. (2018) 131:58–62. 10.1016/j.plaphy.2018.04.033 29735369

[204] Endo A Saika H Takemura M Misawa N Toki S. A novel approach to carotenoid accumulation in rice callus by mimicking the cauliflower Orange mutation via genome editing. Rice. (2019) 12:1–5. 10.1186/s12284-019-0345-3 31713832 PMC6851270

[205] Zhu Y Lin Y Chen S Liu H Chen Z Fan M. CRISPR/Cas9-mediated functional recovery of the recessive rc allele to develop red rice. Plant Biotechnol J. (2019) 17:2096–105. 10.1111/pbi.13125 31002444 PMC6790373

[206] Sánchez-León S Gil-Humanes J Ozuna C V. Giménez MJ Sousa C Voytas DF. Low-gluten, nontransgenic wheat engineered with CRISPR/Cas9. Plant Biotechnol J. (2018) 16:902–10. 10.1111/pbi.12837 28921815 PMC5867031

[207] Liang Z Zhang K Chen K Gao C. Targeted mutagenesis in Zea mays using TALENs and the CRISPR/Cas system. J Genet Genomics. (2014) 41:63–8. 10.1016/j.jgg.2013.12.001 24576457

[208] Subedi U Jayawardhane KN Pan X Ozga J Chen G Foroud NA. The potential of genome editing for improving seed oil content and fatty acid composition in oilseed crops. Lipids. (2020) 55:495–512. 10.1002/lipd.12249 32856292

[209] Li X Wang Y Chen S Tian H Fu D Zhu B. Lycopene is enriched in tomato fruit by CRISPR/Cas9-mediated multiplex genome editing. Front Plant Sci. (2018) 9:559. 10.3389/fpls.2018.00559 29755497 PMC5935052

[210] Nonaka S Arai C Takayama M Matsukura C Ezura H. Efficient increase of Γ-aminobutyric acid (GABA) content in tomato fruits by targeted mutagenesis. Sci Rep. (2017) 7:7057. 10.1038/s41598-017-06400-y 28765632 PMC5539196

[211] Ito Y Nishizawa-Yokoi A Endo M Mikami M Toki S. CRISPR/Cas9-mediated mutagenesis of the RIN locus that regulates tomato fruit ripening. Biochem Biophys Res Commun. (2015) 467:76–82. 10.1016/j.bbrc.2015.09.117 26408904

[212] Li R Fu D Zhu B Luo Y Zhu H. CRISPR/Cas9-mediated mutagenesis of lncRNA1459 alters tomato fruit ripening. Plant J. (2018) 94:513–24. 10.1111/tpj.13872 29446503

[213] Andersson M Turesson H Olsson N Fält AS Ohlsson P Gonzalez MN. Genome editing in potato via CRISPR-Cas9 ribonucleoprotein delivery. Physiol Plant. (2018) 164:378–84. 10.1111/ppl.12731 29572864

[214] González MN Massa GA Andersson M Turesson H Olsson N Fält AS. Reduced enzymatic browning in potato tubers by specific editing of a polyphenol oxidase gene via ribonucleoprotein complexes delivery of the CRISPR/Cas9 system. Front Plant Sci. (2020) 10:1649. 10.3389/fpls.2019.01649 31998338 PMC6962139

[215] Nakayasu M Akiyama R Lee HJ Osakabe K Osakabe Y Watanabe B. Generation of α-solanine-free hairy roots of potato by CRISPR/Cas9 mediated genome editing of the St16DOX gene. Plant Physiol Biochem. (2018) 131:70–77. 10.1016/j.plaphy.2018.04.026 29735370

[216] Sastry KS Zitter TA. Management of virus and viroid diseases of crops in the tropics. In: Sastry KS Zitter TA editors. Plant Virus and Viroid Diseases in the Tropics. Dordrecht: Springer (2014). p. 149–480. 10.1007/978-94-007-7820-7_2

[217] Sharma SK Chanu NT Anand YR Singh YH Singh S Raj C. First report of large cardamom chirke virus, a macluravirus naturally infecting chili crop in India. Plant Dis. (2019) 103:777. 10.1094/PDIS-09-18-1584-PDN30812563

[218] Sidharthan VK Sharma SK Baranwal VK. The first near-complete genome sequence of large cardamom chirke virus mined from the transcriptome dataset of large cardamom. Plant Gene. (2021) 28:100324. 10.1016/j.plgene.2021.100324

[219] Zhang R Liu J Chai Z Chen S Bai Y Zong Y. Generation of herbicide tolerance traits and a new selectable marker in wheat using base editing. Nat Plants. (2019) 5:480–5. 10.1038/s41477-019-0405-0 30988404

[220] Roy A Zhai Y Ortiz J Neff M Mandal B Mukherjee SK. Multiplexed editing of a begomovirus genome restricts escape mutant formation and disease development. PLoS One. (2019) 14:e0223765. 10.1371/journal.pone.0223765 31644604 PMC6808502

[221] Mubarik MS Wang X Khan SH Ahmad A Khan Z Amjid MW. Engineering broad-spectrum resistance to cotton leaf curl disease by CRISPR-Cas9 based multiplex editing in plants. GM Crop Food. (2021) 1–12. 10.1080/21645698.2021.1938488 34124996 PMC9208622

[222] Vu T Van Sivankalyani V Kim EJ Doan DTH Tran MT Kim J. Highly efficient homology-directed repair using CRISPR/Cpf1-geminiviral replicon in tomato. Plant Biotechnol J. (2020) 18:2133–43. 10.1111/pbi.13373 32176419 PMC7540044

[223] Shimatani Z Kashojiya S Takayama M Terada R Arazoe T Ishii H. Targeted base editing in rice and tomato using a CRISPR-Cas9 cytidine deaminase fusion. Nat Biotechnol. (2017) 35:441–3. 10.1038/nbt.3833 28346401

[224] Garcia Ruiz MT Knapp AN Garcia-Ruiz H. Profile of genetically modified plants authorized in Mexico. GM Crop Food. (2018) 9:152–68. 10.1080/21645698.2018.1507601 30388927 PMC6277063

[225] Eckerstorfer MF Engelhard M Heissenberger A Simon S Teichmann H. Plants developed by new genetic modification techniques-Comparison of existing regulatory frameworks in the EU and Non-EU countries. Front Bioeng Biotechnol. (2019) 7:26. 10.3389/fbioe.2019.00026 30838207 PMC6389621

[226] Van Vu T Sung YW Kim J Doan DTH Tran MT Kim JY. Challenges and perspectives in homology-directed gene targeting in monocot plants. Rice. (2019) 12:95. 10.1186/s12284-019-0355-1 31858277 PMC6923311

[227] Ahmad S Shahzad R Jamil S Tabassum J Chaudhary MAM Atif RM. Regulatory aspects, risk assessment, and toxicity associated with RNAi and CRISPR methods. In: Abd-Elsalam KA Lim K-T editors. CRISPR and RNAi Systems. Amsterdam: Elsevier (2021). p. 687–721. 10.1016/B978-0-12-821910-2.00013-8

[228] Genetically-Engineered Crops Past Experience and Future Prospects | National Academies. Available online at: https://www.nationalacademies.org/our-work/genetically-engineered-crops-past-experience-and-future-prospects (accessed November 12, 2021).

[229] Waltz E. CRISPR-edited crops free to enter market, skip regulation. Nat Biotechnol. (2016) 34:582. 10.1038/nbt0616-582 27281401

[230] Schuttelaar Partners. The Regulatory Status of New Breeding Techniques in Countries Outside the European Union. Developed by Schuttelaar & Partners (2015). Available online at: https://www.nbtplatform.org/background-documents/rep-regulatory-status-of-nbts-oustide-the-eu-june-2015 (accessed October 30, 2021).

[231] Spicer A Molnar A. Gene editing of microalgae: scientific progress and regulatory challenges in Europe. Biology. (2018) 7:21. 10.3390/biology7010021 29509719 PMC5872047

[232] Mallapaty S. Australian gene-editing rules adopt ‘middle ground.’ Nature. (2019). 10.1038/d41586-019-01282-8 32317780

[233] Thygesen P. Clarifying the regulation of genome editing in Australia: situation for genetically modified organisms. Transgenic Res. (2019) 28:151–159. 10.1007/s11248-019-00151-4 31321698

[234] Eriksson D. The Swedish policy approach to directed mutagenesis in a European context. Physiol Plant. (2018) 164:385–95. 10.1111/ppl.12740 29602252

