# Supplementary material for: CRISPR-Cas-Led Revolution in Diagnosis and Management of Emerging Plant Viruses: New Avenues Toward Food and Nutritional Security
Source: Front Nutr. 2021 Dec 16;8:751512. doi: 10.3389/fnut.2021.751512 (PMC8716883; doi:10.3389/fnut.2021.751512)
Supplement: Supplementary file 1 [file Table_1.docx]

**SUPPLEMENTARY TABLE 1 | The ratings on the gene-editing index which reflect the current state of gene editing regulations in different countries**

| **Country/Region** | **Food/Crops** | **Ag Rating** |
| --- | --- | --- |
| Brazil | 10 | 10 |
| Argentina | 10 | 10 |
| Paraguay | 10 | 10 |
| Japan | 8 | 8 |
| Canada | 8 | 8 |
| Australia | 8 | 8 |
| Israel | 10 | 7.5 |
| US | 10 | 7 |
| Central America | 6 | 6 |
| Uruguay | 6 | 6 |
| India | 6 | 6 |
| Chile | 10 | 5.5 |
| Colombia | 10 | 5.5 |
| Russia | 5 | 5 |
| China | 5 | 5 |
| New Zealand | 4 | 4 |
| UK | 2 | 2 |
| EU | 2 | 2 |
| Ukraine | 1 | 1 |
| Mexico | 1 | 1 |
| **Colors and Ratings Guide** | | |
| **Regulation Status** | | **Rating** |
| Determined: No Unique Regulations | | 10 |
| Lightly Regulated | | 8 |
| Proposed: No Unique Regulations | | 6 |
| Ongoing Research, Regulations In Development | | 5 |
| Highly Regulated | | 4 |
| Mostly Prohibited | | 2 |
| Limited Research, No Clear Regulations | | 1 |
| Prohibited | | 0 |
